# Pyruvate Dehydrogenase Kinase Is a Metabolic Checkpoint for Polarization of Macrophages to the M1 Phenotype

**DOI:** 10.3389/fimmu.2019.00944

**Published:** 2019-05-07

**Authors:** Byong-Keol Min, Sungmi Park, Hyeon-Ji Kang, Dong Wook Kim, Hye Jin Ham, Chae-Myeong Ha, Byung-Jun Choi, Jung Yi Lee, Chang Joo Oh, Eun Kyung Yoo, Hui Eon Kim, Byung-Gyu Kim, Jae-Han Jeon, Do Young Hyeon, Daehee Hwang, Yong-Hoon Kim, Chul-Ho Lee, Taeho Lee, Jung-whan Kim, Yeon-Kyung Choi, Keun-Gyu Park, Ajay Chawla, Jongsoon Lee, Robert A. Harris, In-Kyu Lee

**Affiliations:** ^1^BK21 Plus KNU Biomedical Convergence Programs, Department of Biomedical Science, Kyungpook National University, Daegu, South Korea; ^2^Research Institute of Aging and Metabolism, Kyungpook National University, Daegu, South Korea; ^3^Leading-Edge Research Center for Drug Discovery and Development for Diabetes and Metabolic Disease, Kyungpook National University Hospital, Daegu, South Korea; ^4^Department of Internal Medicine, School of Medicine, Kyungpook National University, Kyungpook National University Hospital, Daegu, South Korea; ^5^Department of Biological Sciences, Seoul National University, Seoul, South Korea; ^6^Center for Plant Aging Research, Institute for Basic Science, Daegu Gyeongbuk Institute of Science and Technology, Daegu, South Korea; ^7^Laboratory Animal Resource Center, Korea Research Institute of Bioscience and Biotechnology, Daejeon, South Korea; ^8^College of Pharmacy, Kyungpook National University, Daegu, South Korea; ^9^Department of Biological Sciences, The University of Texas at Dallas, Richardson, TX, United States; ^10^Department of Medicine, University of California, San Francisco, San Francisco, CA, United States; ^11^Soonchunhyang Institute of Medi-Bio Science, Soon Chun Hyang University, Cheonan, South Korea; ^12^Department of Biochemistry and Molecular Biology, Indiana University School of Medicine, Indianapolis, IN, United States

**Keywords:** dichloroacetate, high-fat diet, inflammation, insulin resistance, macrophage polarization, metabolic reprogramming, pyruvate dehydrogenase kinase

## Abstract

Metabolic reprogramming during macrophage polarization supports the effector functions of these cells in health and disease. Here, we demonstrate that pyruvate dehydrogenase kinase (PDK), which inhibits the pyruvate dehydrogenase-mediated conversion of cytosolic pyruvate to mitochondrial acetyl-CoA, functions as a metabolic checkpoint in M1 macrophages. Polarization was not prevented by PDK2 or PDK4 deletion but was fully prevented by the combined deletion of PDK2 and PDK4; this lack of polarization was correlated with improved mitochondrial respiration and rewiring of metabolic breaks that are characterized by increased glycolytic intermediates and reduced metabolites in the TCA cycle. Genetic deletion or pharmacological inhibition of PDK2/4 prevents polarization of macrophages to the M1 phenotype in response to inflammatory stimuli (lipopolysaccharide plus IFN-γ). Transplantation of PDK2/4-deficient bone marrow into irradiated wild-type mice to produce mice with PDK2/4-deficient myeloid cells prevented M1 polarization, reduced obesity-associated insulin resistance, and ameliorated adipose tissue inflammation. A novel, pharmacological PDK inhibitor, KPLH1130, improved high-fat diet-induced insulin resistance; this was correlated with a reduction in the levels of pro-inflammatory markers and improved mitochondrial function. These studies identify PDK2/4 as a metabolic checkpoint for M1 phenotype polarization of macrophages, which could potentially be exploited as a novel therapeutic target for obesity-associated metabolic disorders and other inflammatory conditions.

## Introduction

Macrophage polarization (M1/M2) requires metabolic reprogramming that enhances glycolysis and repurposes mitochondrial function (1–3). Although the importance of these metabolic pathway differences between M1 and M2 macrophages is well-established, our knowledge of the checkpoints in affected metabolic pathways is limited primarily to HIF-1α and pyruvate kinase M2 (PKM2) (4). Obesity-induced insulin resistance is a disease process in which M1 macrophages contribute to adipose tissue (AT) inflammation and insulin resistance. Chronic low-grade inflammation in multiple organs increases the risk of developing obesity, diabetes, cardiovascular diseases, and cancers, indicating a major role for the immune system in the etiology of metabolic disorders (5). Recruitment of M1 macrophages, IFN-γ-secreting Th1 cells, CD8^+^ T cells, and B cells in the adipose tissue drives the inflammatory response, locally promoting systemic inflammation and impaired insulin action as a result of over-nutrition (6, 7). The phenotypic changes in macrophages that occur in response to over consumption of energy are considered potential therapeutic targets for managing chronic metabolic diseases.

Pyruvate dehydrogenase kinase (PDK) provides a therapeutic target for the Warburg effect in malignant cancers (8) and has been suggested to serve this function during macrophage polarization (9). PDK1 participates in M1 macrophage polarization via HIF-1α-mediated aerobic glycolysis, accounting for the proinflammatory responses (9). In contrast, among the four PDK isozymes, PDK2 and PDK4 are the most strongly associated with metabolic diseases, especially type 2-diabetes (10). Recent work from our laboratory has indicated that dual deficiency of *Pdk2* and *Pdk4* (PDK2/4 DKO) attenuates the lactic acid surge, the proinflammatory markers, and the pain hypersensitivity suggesting a key role for the PDK-PDH-lactic acid axis in the pathogenesis of inflammatory pain mediated by macrophage functional regulation (11). This finding suggests a novel therapeutic approach for many inflammatory conditions but is seemingly at odds with conclusions of others who have addressed the role of the PDKs in macrophage polarization (12–14); we have, therefore, examined this phenomenon in greater depth in the present study.

Here we provide additional evidence for PDK4 induction in macrophages in response to LPS and IFN-γ. We also show that genetic and pharmacological blockage of PDK activity in mice fed a high-fat diet (HFD) represses macrophage M1 polarization, which is correlated with amelioration of adipose tissue inflammation as well as insulin resistance. These findings support the hypothesis that the PDKs are therapeutic targets for inflammatory diseases.

## Materials and Methods

### Animals

All experiments were approved by the Institutional Animal Care and Use Committee of Kyungpook National University and were conducted according to recommendations in the National Institutes of Health Guide for the Care and Use of Laboratory Animals. Eight-week-old male WT (C57BL/6J) and PDK2/4 DKO mice (15) were either fed a HFD 20% of the calories derived from carbohydrates and 60% from fat (Research Diets; D12492 pellets) for use as a diet-induced obesity (DIO) model or fed an isocaloric control diet (CD) in which 70% of the calories were derived from carbohydrates and 10% from fat (Research Diets; D12450B pellets). The mice were housed and maintained on a 12 h light/dark cycle at 22 ± 2°C. After the mice were sacrificed, the tissues were rapidly collected and freeze-clamped with liquid nitrogen-cooled Wollenberger tongs and stored at −80°C prior to analysis.

### Isolation of Peritoneal Macrophages (PMs)

Eight- to ten-week-old WT, PDK2 KO (2KO), PDK4 KO (4KO), and PDK2/4 DKO mice (15–17) were injected with 3% thioglycollate broth, i.p., and then sacrificed 4 days later. Peritoneal lavage was performed twice using 4 mL of 1X PBS, and the harvested cells were then cultured in RPMI1640 (Gibco; 11875-093) supplemented with antibiotics. After 1 h of culture, the suspended cells were discarded, and the adherent cells were used for experiments.

### Isolation and Differentiation of Bone Marrow-Derived Macrophages (BMDMs)

Bone marrow cells were collected from the femurs and tibias of 8- to 10-week-old mice. The cells were cultured at 2 × 10^7^ cells/plate in α-MEM medium (WELGENE; LM 008-02) containing 30% L929-conditioned media and 10% FBS for 9 days to allow the differentiation. The established BMDMs were then used for experiments.

### Isolation of Peritoneal Macrophages (PMs) by Zymosan a or LPS Treatment

Zymosan-elicited peritoneal macrophages (ZEPMs) and LPS-elicited peritoneal macrophages (LEPMs) were isolated as previously reported (18). Briefly, zymosan A (1 mg/mouse) or LPS (1 mg/kg) were i.p. injected into 8-week-old C57BL/6J mice. One day after the injection, peritoneal fluid was harvested and cells were cultured in RPMI1640 supplemented with antibiotics. After 1 h of culture, the suspended cells were discarded and the adherent cells were used for experiments.

### Western Blot Analysis

The tissue cells were lysed using a lysis buffer [20 mM Tris (pH 7.4), 10 mM Na_4_P_2_OH, 100 mM NaF, 2 mM Na_3_VO_4_, 5 mM EDTA (pH 8.0), 0.1 mM PMSF, and 1% NP-40] containing protease inhibitors (aprotinin 7 μg/mL and leupeptin 7 μg/mL) and phosphatase inhibitor cocktail. Protein concentrations were measured using BCA protein assay reagent (Thermo Fisher Scientific; 23225). Cell lysates were separated on 10% SDS-polyacrylamide gels and then transferred to polyvinylidene difluoride membranes (Merck Millipore; IPVH00010). The transferred proteins on the membrane were immunoblotted with the following primary antibodies: anti-HIF-1α (1:1,000), anti-iNOS (1:1,000), anti-Arg-1 (1:1,000), anti-PDK2 (1:1,000), anti-PDK4 (1:500), and anti-β-actin (1:5,000). All antibodies were diluted in TBST containing 5% BSA.

### Measurement of Oxygen Consumption Rate (OCR)

The OCR was measured using a Seahorse XF-24 Flux Analyzer (Seahorse Biosciences, Billerica, MA, USA). BMDMs were seeded in XF-24 tissue culture plates (24-well) at a density of 1 × 10^5^ cells/well and incubated overnight. The cells were treated with M1 stimulants (LPS 100 ng/mL + IFN-γ 10 ng/mL) with or without dichloroacetate (DCA 10 mM, Sigma; 47795) for 3 h. The assay medium used consisted of XF base medium (Seahorse Biosciences) supplemented with 5.5 mM D-glucose (Sigma-Aldrich; G7528), 1 mM sodium pyruvate (Sigma-Aldrich; S8636), and 1X GlutaMAX™ (Gibco; 35050) and adjusted to pH 7.4. The inhibitors and uncouplers used in this study were as follows: oligomycin A (2 μM, Sigma-Aldrich; 75351), CCCP (carbonyl cyanide 3-chlorophenylhydrazone, 7.5 μM, Sigma-Aldrich; C2759), rotenone (1 μM, Sigma-Aldrich; R8875), and antimycin A (2.5 μM, Sigma-Aldrich; A8674). OCR was normalized to protein concentration.

### Metabolite Extraction

Isolated cells were cultured overnight in 10% dialyzed FBS media. The medium was replaced with fresh 10% dialyzed FBS medium and cells were then stimulated with LPS (100 ng/mL) and IFN-γ (10 ng/mL) for 12 h. Cells were washed with 3 mL ice-cold 0.9% NaCl twice and then collected in Eppendorf tubes. Cells were resuspended in 200 μL of ice-cold metabolite extraction solution (chloroform:methanol:water 1:3:1, v/v) and then sonicated. After incubation on ice for 1 h, the metabolite samples were collected by centrifugation at 13,000 rpm for 5 min. All the samples were lyophilized and re-suspended in 300 μL of water containing 0.1% formic acid, prior to LC-MS/MS analysis.

### Data Presentation and Statistical Analysis

Data were presented using the GraphPad Prism software and statistical analysis was performed using IBM SPSS Statistics (version 21). Statistically significant differences were measured by Student's *t*-test for normally distributed data. Statistical analysis of group comparison was performed by one-way or two-way ANOVA followed by Tukey's HSD (honestly significant difference) *post hoc* test. *p*-values < 0.05 were considered statistically significant. Detailed procedures are included as part of Supplemental Materials.

### Detailed Procedures

See the Supplemental Materials.

## Results

### PDK2 and PDK4 Are Required for M1 Macrophage Polarization

**Figure 1:**
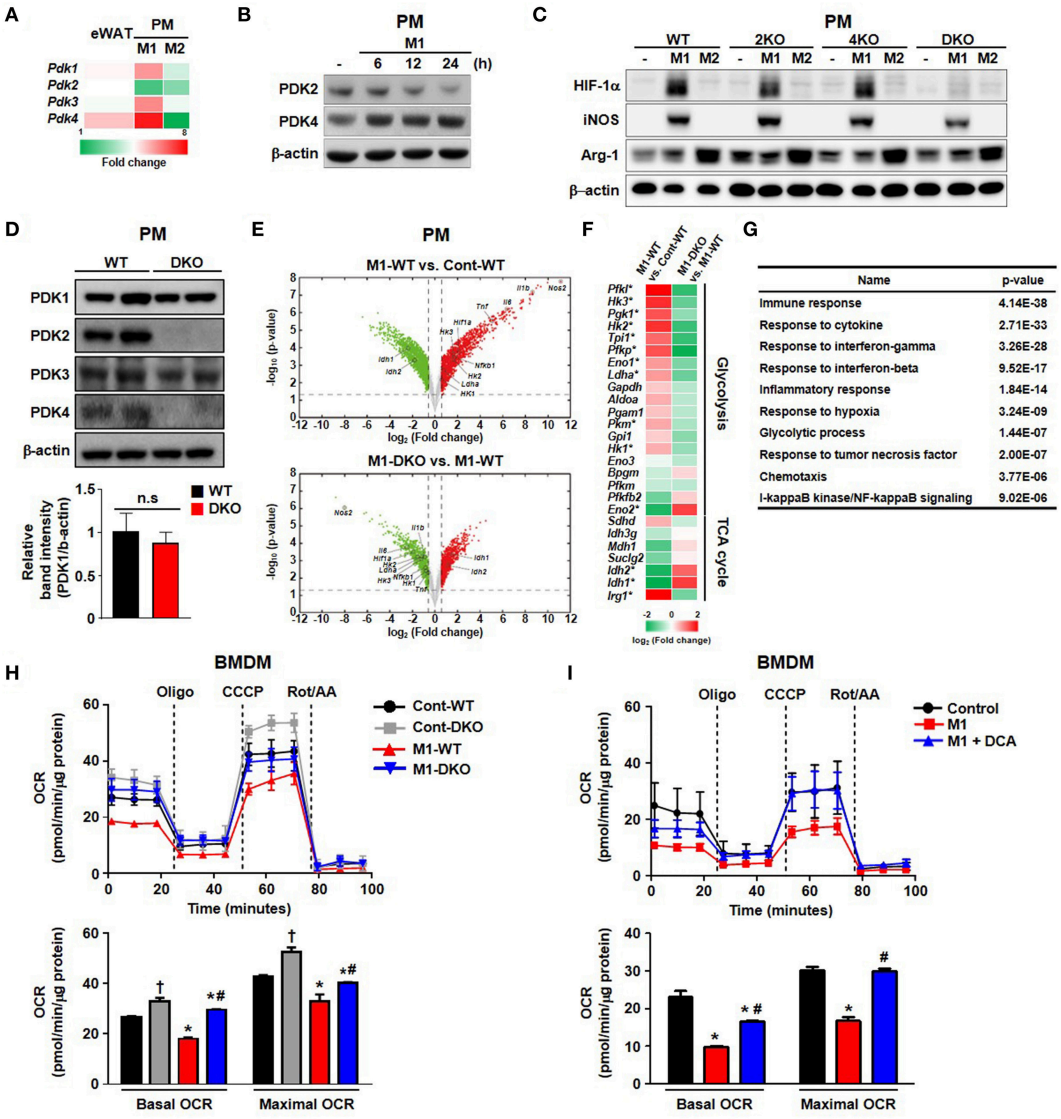
PDK2 and PDK4 are required for M1 macrophage polarization. **(A)** Heat map for relative mRNA levels of PDK isoforms measured by qRT-PCR. eWAT refers to epididymal AT from WT mice fed an HFD in comparison to epididymal AT from WT mice fed a CD for 24 weeks. M1 refers to PM stimulated for 24 h with LPS 100 ng/mL+ IFN-γ 10 ng/mL relative to unstimulated PM. M2 refers to PM stimulated for 24 h with IL-4 10 ng/mL+ IL-10 10 ng/mL relative to unstimulated PMs. *n* = 5–7 per group for AT and *n* = 3 per group for PMs. Values are expressed as mean ± SD for PMs and ± SEM for AT. Statistical analysis was performed by Student's *t*-test. ^*^p < 0.05 vs. control. **(B)** PDK expression levels were assessed in PMs after M1 stimulation with LPS 100 ng/mL+ IFN-γ 10 ng/mL for the indicated times. **(C)** M1 and M2 markers in PMs prepared from WT, PDK2 KO, PDK4 KO, and PDK2/4 DKO mice followed by M1 stimulation with LPS 100 ng/mL+ IFN-γ 10 ng/mL or M2 stimulation with IL-4 10 ng/mL+ IL-10 10 ng/mL for 12 h. **(D)** The expressions of PDK isozymes in PMs from WT and DKO mice. **(E)** Volcano plots for the comparisons of M1-WT vs. Cont-WT (upper) and M1-DKO vs. M1-WT (lower). Dotted lines represent the cutoffs for fold change and *p*-value. Red and green dots denote up- and down-regulated genes, respectively. **(F)** Heat map of genes involved in glycolysis and TCA cycle. The color bar represents the gradient of log2 (fold change) of mRNA expression levels in each comparison. The genes that were up- (down-) regulated in M1-WT compared to Cont-WT and down- (up-) regulated in M1-DKO compared to M1-WT at the same time were indicated by an asterisk. **(G)** GOBPs represented by the genes that were up-regulated in M1-WT compared to Cont-WT and down-regulated in M1-DKO compared to M1-WT at the same time. The p-value is the significance of GOBPs being enriched by the genes. **(H,I)** OCRs were measured in WT- or DKO-BMDM in the presence or absence of M1 stimulation by LPS (100 ng/mL) + IFN- γ (10 ng/mL) treatment for 3 h **(H)** and DCA (10 mM) treatment **(I)**; *n* = 4–5 per group. Values are expressed as mean ± SD. Statistical analysis was performed by Student's *t*-test. ^*^p < 0.05 vs. (–) M1 stimulation, ^†^*p* < 0.05 vs. control-WT, and #*p* < 0.05 vs. M1-WT.

We explored the role of PDKs in overnutrition-induced AT inflammation. Among the 4 different isoforms of PDK, the mRNA expression of only *Pdk4* was significantly upregulated in the AT of mice fed an HFD compared to those fed a CD (Figure 1A). Furthermore, *Pdk4* was more responsive to specific M1 stimulation, which was correlated with the upregulation of *Pdk4*, and M2 stimulation, correlated with *pdk4* downregulation in macrophages (Figures 1A,B and Figures S1A–D). The induction of HIF-1α and iNOS by M1-only stimulation and M1+M2 stimulation, respectively, to mimic *in vivo* wild-type (WT) conditions, was completely suppressed in PDK2/4 DKO mice but only slightly suppressed in PDK2 or PDK4 KO-peritoneal macrophages (PMs) and bone marrow-derived macrophages (BMDMs) (Figure 1C), suggesting that PDK2 and PDK4 can functionally compensate for each other. And we confirmed no change of PDK1 expression in DKO mice (Figure 1D and Figures S1E,F). Conversely, M2 stimulation with IL-4 caused a greater increase in arginase-1 expression in a time-dependent manner in PMs from PDK2/4 DKO mice compared to those from WT mice (Figure S1G). Since HIF-1α is essential for the upregulation of glycolytic genes and, therefore, the activation of inflammatory macrophages, suppression of the increase in HIF-1α by PDK2/4 deficiency in response to LPS + IFN-γ stimulation is especially noteworthy.

RNA-seq analysis revealed increased aerobic glycolysis in response to M1 stimulation; this was indicated by increased expression of glycolysis-related genes and decreased expression of TCA cycle-related genes. This response was practically absent in LPS + IFN-γ-stimulated PMs from PDK2/4 DKO mice (Figures 1E–G). Consistently, the decrease in isocitrate dehydrogenase (*Idh*)1 and *Idh*2 mRNA expression levels that normally occur in response to LPS + IFN-γ stimulation was prevented (Figure 1F). The expression of *Irg1* required for the production of itaconic acid, an important factor in the antimicrobial response of macrophages was less affected, yet significantly suppressed in the PMs from PDK2/4 DKO mice (Figure 1F). To evaluate the mitochondrial function in response to reduced PDK activity, we measured the oxygen consumption rate (OCR) in both PDK2/4-deficient BMDMs and dichloroacetate (DCA; pan PDK inhibitor)-treated BMDMs. M1 polarization significantly reduced basal and maximal OCR in WT-BMDMs; however, the OCR in LPS + IFN-γ treated PDK2/4 DKO-BMDMs was still comparable to that in WT-BMDMs (M0) (Figure 1H). Likewise, DCA treatment was found to largely prevent the reduction in basal and maximal OCR in LPS + IFN-γ-treated WT-BMDMs, which are known, otherwise, to have a broken TCA cycle (evidenced by the accumulation of succinate) during M1 polarization (Figure 1I) (19). Elevated extracellular lactate and intracellular succinate levels were also significantly attenuated in PDK2/4 DKO-BMDMs compared to that in WT-BMDMs (Figures S2A,B). Overall, our data are indicative that genetic as well as pharmacological inhibition of PDK2/4 can block the metabolic reprogramming of aerobic glycolysis and mitochondrial respiration under conditions that normally induce M1 macrophage polarization.

### PDK2/4 Deficiency Prevents the Increase in Glycolytic Intermediates and the Decrease in TCA Cycle Intermediates Normally Induced by Treating Macrophages With Inflammatory Stimuli

**Figure 2:**
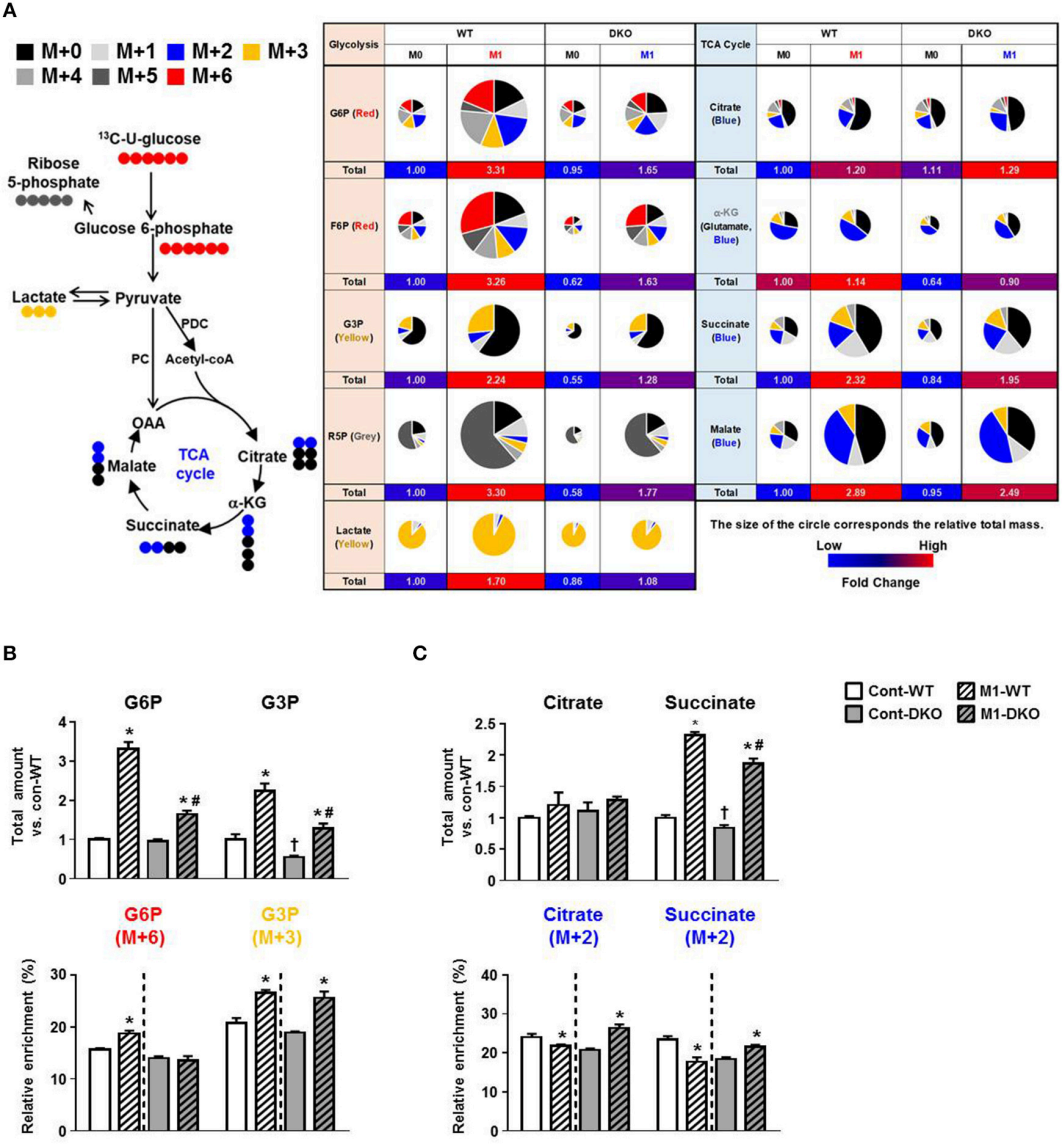
PDK2/4 deletion increases glucose oxidation by macrophages. **(A)** Schematic representation of metabolite labeling by ^13^C_6_-glucose. The relative amounts of metabolites are represented by the pie charts and the different colors indicate labeling with different mass isotopomers. **(B,C)** The relative amounts and ^13^C enrichment patterns of the intermediates of the glycolytic **(B)** and TCA cycle **(C)** in WT- and DKO-PMs compared with control-WT; *n* = 4–5 per group. Values are expressed as mean ± standard error of mean (SEM). Statistical analysis was performed by Student's *t* test. ^*^p < 0.05 vs. (-) M1 stimulation, ^†^*p* < 0.05 vs. control-WT, and # *p* < 0.05 vs. M1-WT.

Based on our finding that mitochondrial respiration is enhanced when M1 polarization is prevented by PDK2/4-deficiency, we hypothesized that acetyl-CoA (generated by conversion of pyruvate to acetyl-CoA by PDH) might be utilized efficiently by the intermediates of TCA cycle, resulting in increased OXPHOS with less lactate production. To test our hypothesis, we performed ^13^C_6_-glucose trace analysis (a schematic representation of metabolite labeling with ^13^C_6_-glucose is given in Figure 2A) and assessed the relative mass distribution vector represented by the metabolites derived from ^13^C_6_-glucose directly, as well as the total amount of metabolites from glucose metabolism during PDK2/4 deficiency (Figures 2B,C) (20). As assessed by two different methods and consistent with the altered expression of glycolytic enzymes (Figure 1F), the steady state amounts of metabolites between glucose to lactate were significantly increased in M1-polarized WT-PMs, while PDK2/4 deficiency was found to prevent increases in these intermediates (Figure 2B, Figure S2C). Although the mass of citrate was not significantly affected, formation of M+2 citrate (blue in the pie chart) from M+6 glucose was reduced in M1-polarized WT-PM but increased by PDK2/4 deficiency, consistent with decreased flux in the former and increased flux in the latter (Figure 2C). Although total amounts of succinate and malate were significantly increased, M+2 succinate and M+2 malate levels (blue in the pie chart) were significantly reduced in M1-polarized WT-PM. Interestingly, metabolic flux analysis of these intermediates displayed enhanced enrichments in PDK2/4-deficient conditions (Figure 2C; Figures S2D,E). These data suggest that increased provision of acetyl-CoA for the maintenance of the TCA cycle by PDK2/4 deficiency prevents the decrease in cellular respiration characteristic of M1-polarized WT macrophages.

### Global PDK 2/4 Deficiency in HFD-Fed Mice Reduces Insulin Resistance and AT Inflammation

**Figure 3:**
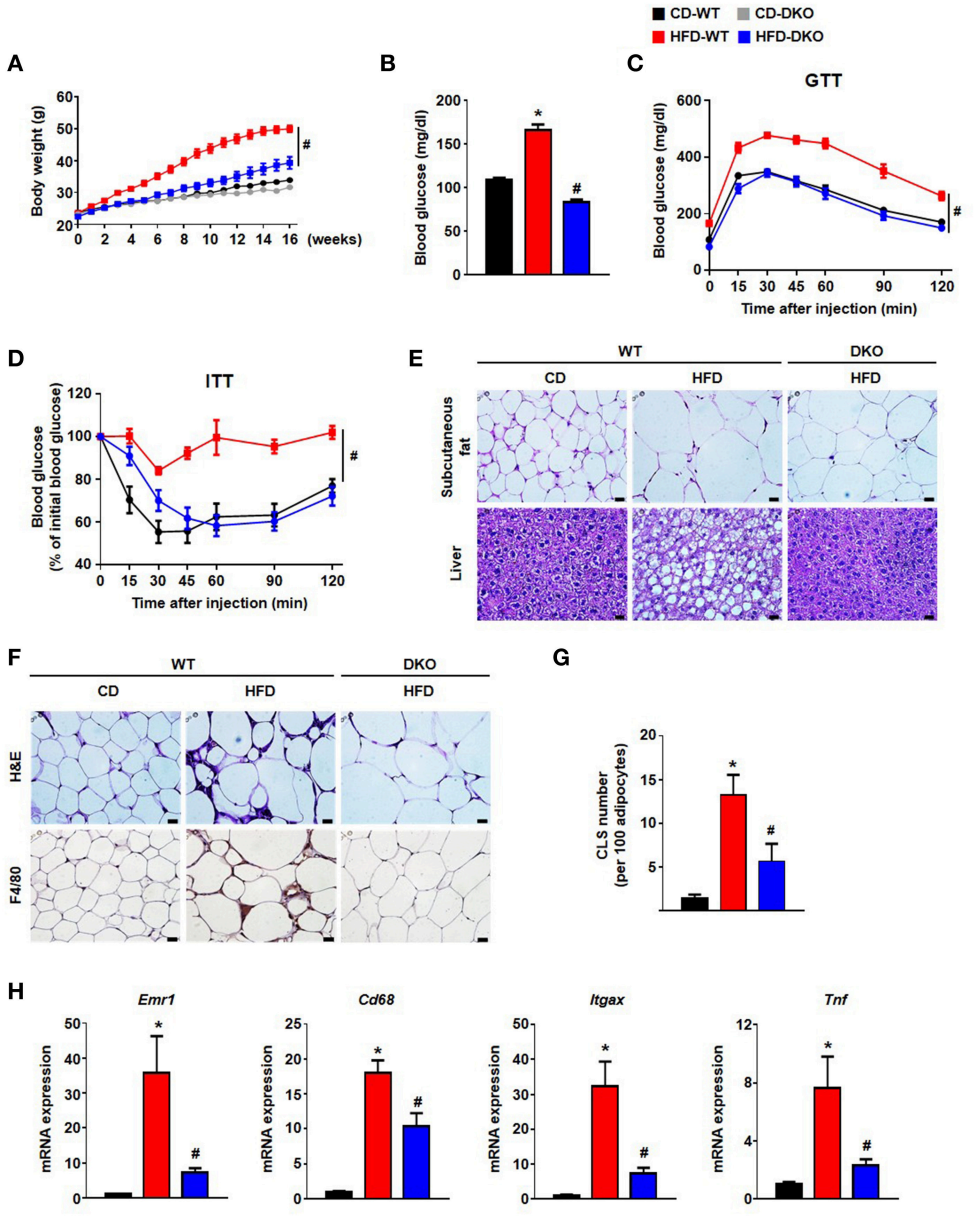
PDK2/4 deficiency ameliorates adipose tissue inflammation and insulin resistance in mice fed a high fat diet. **(A–D)** Measurements of body weights **(A)**, fasting blood glucose levels **(B)**, glucose tolerance test (1.5 g/kg, i.p.) after 16 h fasting **(C)** and insulin tolerance test (0.75 U/kg, i.p.) after 6 h fasting **(D)** were performed in CD or HFD-fed mice; *n* = 8–14 per group. **(E)** Representative morphology of the subcutaneous AT and liver from mice fed CD or HFD for 24 weeks, as shown by H&E staining; magnification: 400X; scale bar: 20 μm. **(F,G)** Representative morphology of the epididymal AT, as shown by H&E staining or IHC staining for F4/80; magnification: 400X; scale bar: 20 μm **(F)**. Quantification of crown-like structures in the epididymal AT; *n* = 6–9 per group **(G)**. **(H)** mRNA expression levels of inflammatory genes in the epididymal AT; *n* = 7–12 per group. Values are expressed as mean ± SEM. Statistical analysis was performed by Student's *t-*test. ^*^p < 0.05 vs. CD-WT, and #*p* < 0.05 vs. HFD-WT.

Greater PDH activity caused by global PDK 2/4 deficiency improves insulin sensitivity (15), suggesting PDKs are involved in the development of whole-body insulin resistance. To determine whether DKO mice show reduced HFD-induced AT inflammation which may contribute to the healthier metabolic phenotype of these mice, WT and DKO mice were fed CD or HFD. As reported previously (15), HFD-fed-PDK2/4 ablated mice displayed lower body weight gain, lower fasting blood glucose levels, improved glucose tolerance, and increased insulin sensitivity along with reduced fat accumulation in the AT and liver (Figures 3A–E). Furthermore, the number of infiltrated epididymal AT macrophages was reduced, as indicated by reduction in crown-like structures and the levels of proinflammatory markers Emr1, Cd68, Itgax, and Tnf (Figures 3F–H). These data show that global PDK2/4 deficiency attenuates HFD-induced macrophage infiltration and thereby reduces AT inflammation, suggesting that inhibition of PDH activity by the PDKs is involved in the AT inflammation caused by obesity.

### Bone Marrow-Specific PDK2/4 Deletion Interferes With HFD-Induced AT Macrophage Infiltration

**Figure 4:**
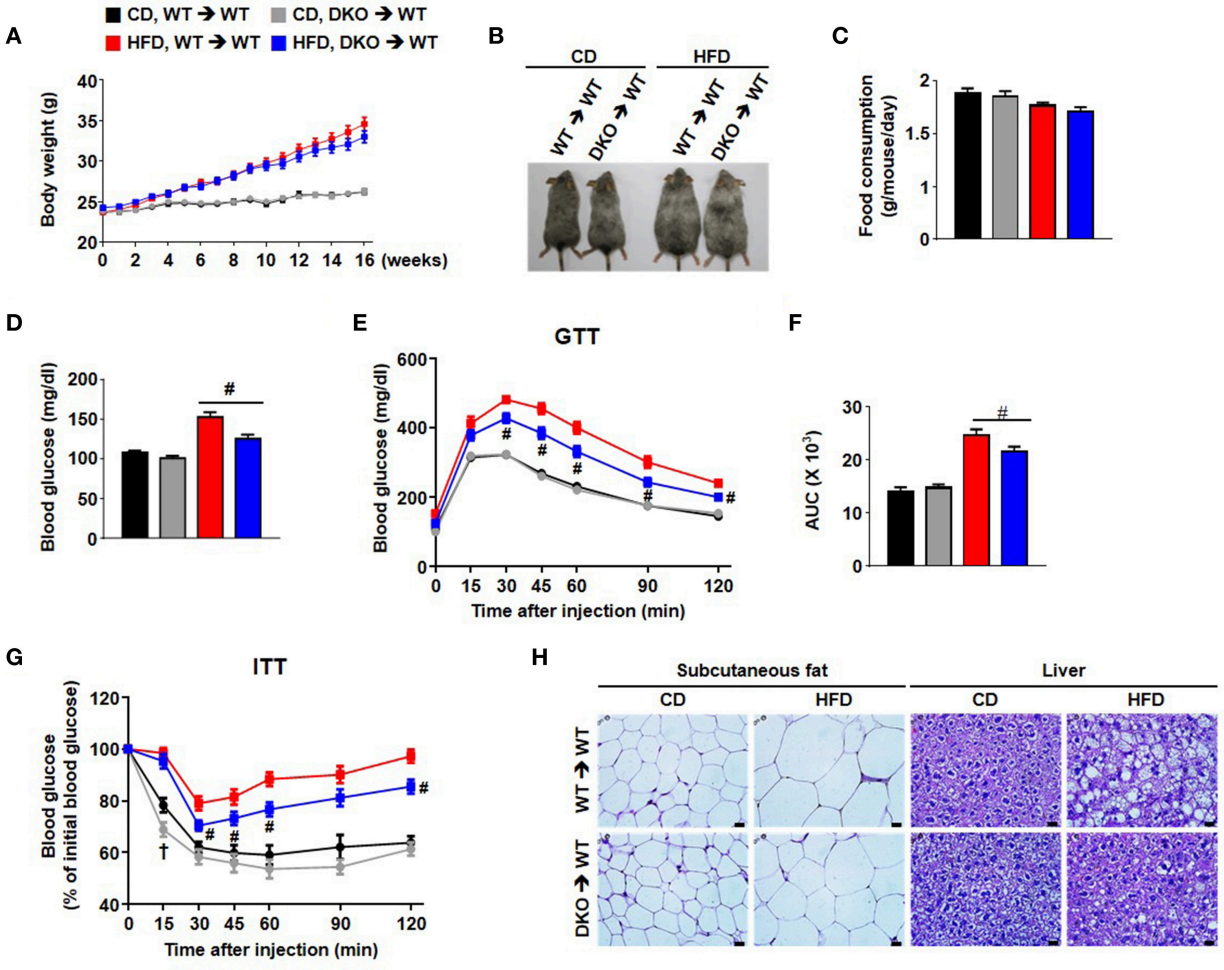
Hematopoietic cell-specific PDK2/4 deficiency attenuates insulin resistance in mice fed a high-fat diet. **(A)** Body weights were measured during feeding of the different diets; *n* = 12–26 per group. **(B)** Representative mice of each group **(C)** Food consumption was measured per day in each group; *n* = 7 per group. **(D–G)** Measurement of fasting blood glucose levels **(D)**, glucose tolerance test (1.5 g/kg, i.p.) after 16 h fasting **(E,F)**, and insulin tolerance test (0.75 U/kg, i.p.) after 6 h fasting **(G)** was performed in both CD and HFD-fed mice; *n* = 11–26 per group. Values are expressed as mean ± SEM. Statistical analysis was performed by one-way or two-way ANOVA followed by Tukey's HSD. # *p* < 0.05 vs. HFD-WT-BMT. **(H)** Representative morphology of the subcutaneous AT and liver from WT- and DKO-BMT mice as shown by H&E staining; magnification: 400X; scale bar: 20 μm.

**Figure 5:**
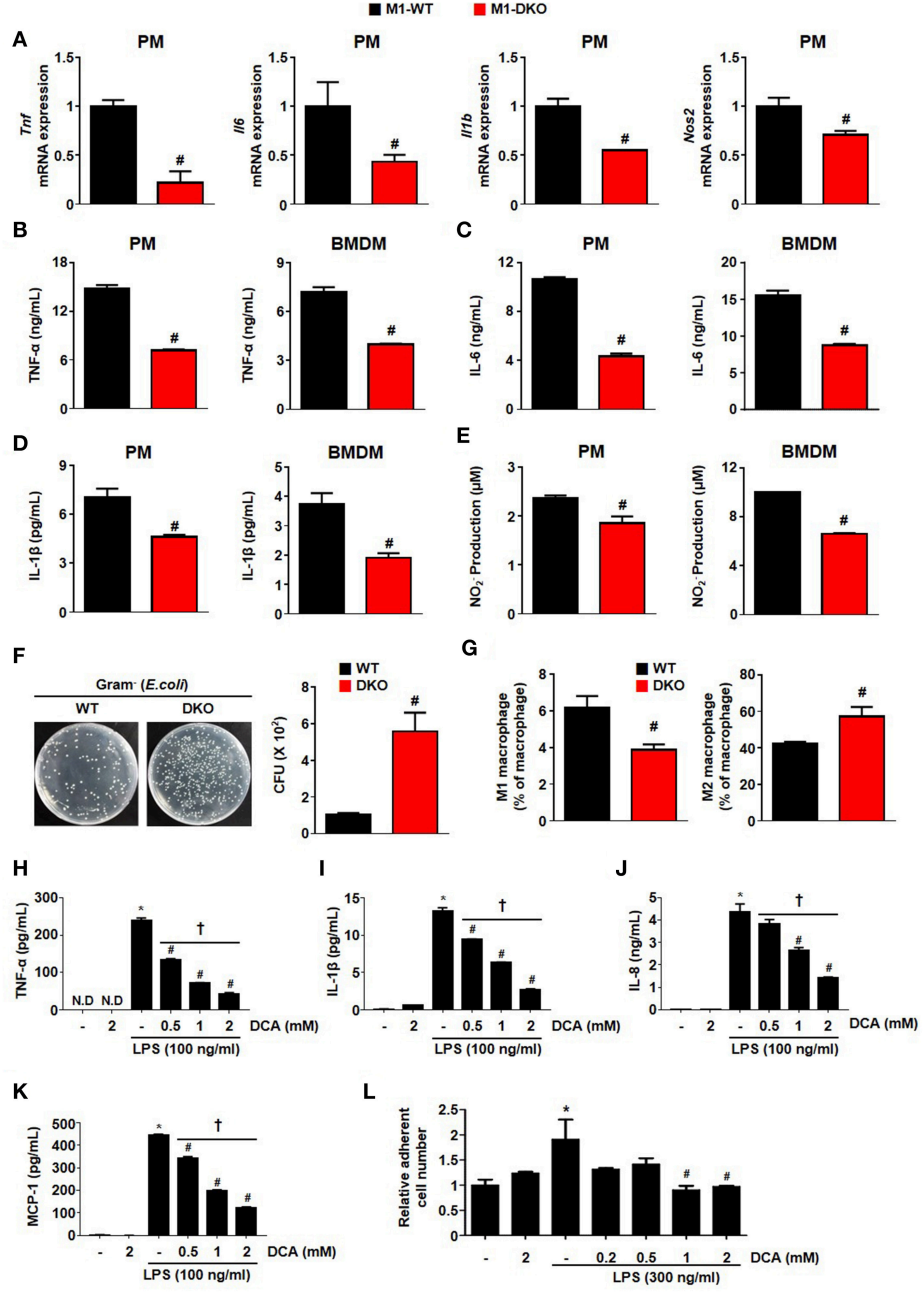
Suppression of PDK2/4 in macrophages attenuates the secretion of proinflammatory effectors in response to inflammatory stimuli. **(A–E)** The levels of mRNA expression **(A)** and secreted proinflammatory effectors **(B–E)** were measured after treatment of WT- and DKO-PMs or -BMDMs with LPS 100 ng/mL+ IFN- γ 10 ng/mL for 12 h; *n* = 3–4 per group. **(F)** Bactericidal effect was assessed by counting the colony numbers in WT- and DKO-PMs; *n* = 5 per group. **(G)** Effect on PDK2/4 deficiency on the *in vivo* macrophage population in LPS (1 mg/kg)-injected mice; *n* = 5 per group. Values are expressed as mean ± SEM. Statistical analysis was performed using Student's *t*-test. #*p* < 0.05 vs. WT. **(H–K)** Secreted proinflammatory cytokine levels were determined at different DCA concentrations in THP1 cells treated with LPS 100 ng/mL for 24 h; *n* = 3 per group. **(L)** Adhesion assay was performed in collagen (10 μg/mL)-coated wells with DCA-treated THP-1 cells incubated with LPS (300 ng/mL) for 24 h; *n* = 3 per group. Values are expressed as mean ± SD. Statistical analysis was performed using Student's *t*-test. ^*^p < 0.05 vs. (–) LPS, #*p* < 0.05 vs. only LPS, and ^†^*p* < 0.05 vs. 2 mM DCA. N.D, not detected.

To investigate the potential effects of innate immune cells on chronic inflammation, a bone-marrow (BM) transplantation mouse model was used to evaluate the direct contribution of BM-derived immune cells from PDK2/4 DKO mice. The method involved irradiation of recipient WT mice to ablate BM cells followed by transplantation via intravenous infusion of donor BM obtained from WT mice or PDK2/4 DKO mice (21); donor as well as recipient mice were maintained on a CD for this study. Four weeks after maintaining all of the mice on a CD, the mice were divided into four groups: 1) WT mice with WT BM that were continued on the CD; 2) WT mice with WT BM that were placed on the HFD; 3) WT mice with DKO BM that were continued on the CD; and 4) WT mice with DKO BM that were placed on the HFD. No differences in any subsequent measurements were observed between the two groups of mice (1 and 3) that were continued on the CD (Figure 4). Likewise, no differences in body weight, body size, and food consumption were observed between the two groups of mice (2 and 4) that were maintained on the HFD (Figures 4A–C). However, remarkable differences in markers of metabolic dysfunction were observed between the latter two groups of mice. The HFD-fed mice transplanted with WT-BM displayed an increase in fasting blood glucose, AT, and liver fat, while the glucose tolerance and insulin sensitivity was found to be reduced. All of these expected negative consequences of HFD feeding were attenuated in mice transplanted with DKO-BM (Figures 4D–H). These findings along with reduced inflammatory responses and no additional anti-inflammatory cytokine production such as IL-10 and TGF-β by PDK2/4 deficiency under sterile inflammatory condition (Figure 5 and Figure S3) are consistent with other reports that have demonstrated improved insulin sensitivity in response to global PDK 2/4 deficiency and, consequently, greater PDH activity (22).

**Figure 6:**
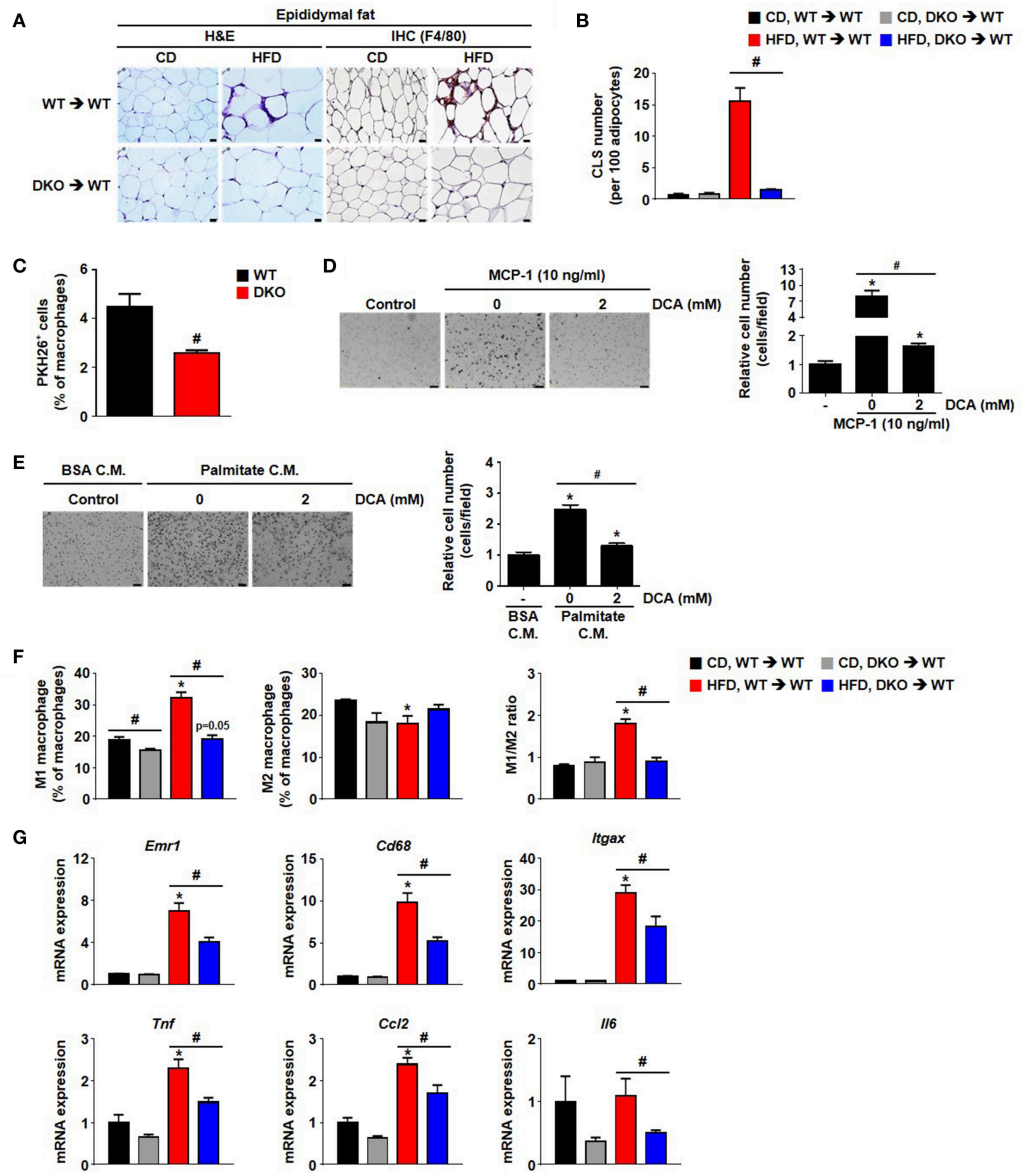
Hematopoietic cell-specific PDK2/4 deficiency attenuates adipose tissue inflammation by preventing the recruitment of M1 macrophages. **(A,B)** Representative morphology of the epididymal AT by H&E staining or IHC staining for F4/80; magnification: 400X; scale bar: 20 μm **(A)**. The numbers of crown-like structures in the epididymal AT were determined; *n* = 8 per group **(B)**. Values are expressed as mean ± SEM. Statistical analysis was performed by one-way ANOVA followed by Tukey's HSD. #*p* < 0.05 vs. HFD-WT-BMT. **(C)** PKH26^+^ cell population was measured by FACS analysis; *n* = 4 per group. Values are expressed as mean ± SEM. Statistical analysis was performed by Student's *t*-test. #*p* < 0.05 vs. WT monocyte donor group. **(D)** MCP-1-induced migration was evaluated in DCA-treated BMDMs using a trans-well migration assay; *n* = 3 per group; magnification: 200X; scale bar: 50 μm. The relative number of migrated cells per field was counted. Values are expressed as mean ± SEM. Statistical analysis was performed by Student's *t-*test. ^*^p < 0.05 vs. control, #*p* < 0.05 vs. MCP-1 induction. **(E)** 3T3-L1-conditioned medium (C.M.)-induced migration was evaluated in DCA-treated BMDMs using a trans-well migration assay; *n* = 3 per group; magnification: 200X; scale bar: 50 μm. The relative number of migrated cells per field was counted. Values are expressed as mean ± SEM. Statistical analysis was performed by Student's *t*-test. ^*^p < 0.05 vs. control, #*p* < 0.05 vs. palmitate C.M. **(F)** M1 and M2 macrophage populations and their ratios were determined in the stromal vascular fraction of WT- and DKO-BMT; *n* = 3 per group. Values are expressed as mean ± SEM. Statistical analysis was performed by Student's *t*-test. ^*^p < 0.05 vs. corresponding CD-fed group, # *p* < 0.05 compared with WT- and DKO-BM donor group. **(G)** mRNA expression levels of inflammatory genes in the epididymal AT; *n* = 7–12 per group. Values are expressed as mean ± SEM. Statistical analysis was performed by Student's *t*-test. ^*^p < 0.05 vs. CD, #*p* < 0.05 vs. WT-BMT.

Furthermore, the number of crown-like structures was dramatically increased in the AT of mice with WT BM but not in the mice with DKO-BM (Figures 6A,B). To confirm the reduced migrating capacity of PDK2/4 ablated monocyte to fat tissue *in vivo*, we assessed that the PKH26-positive stained macrophage (F4/80^+^CD11b^+^PKH26^+^) population derived from DKO monocyte was significantly reduced in the stromal vascular fractions of 4 week HFD mice compared to WT monocyte (Figure 6C). Pharmacological inhibition of PDK activity with DCA was found to significantly attenuate MCP-1-induced migration (Figure 6D). To mimic the *in vivo* physiological effect of HFD, BMDMs were incubated with palmitate-treated, 3T3-L1-conditioned medium. Interestingly, DCA treatment was found to significantly reduce the migration induced by an unknown chemoattractant in palmitate-treated 3T3-L1 cells (Figure 6E). We also confirmed that the increase in M1/M2 ratio as well as levels of proinflammatory markers was significantly blocked in the epididymal AT from WT mice with DKO-BM (Figures 6F,G and Figure S4). These results are indicative that PDK2/4 deletion and PDK inhibition prevent macrophage polarization and infiltration, thereby preventing inflammation of adipose tissue in response to over consumption of dietary energy.

### A Novel PDK Inhibitor Prevents Polarization of Macrophages to the M1 Phenotype and Attenuates Adipose Tissue Inflammation in Obesity

**Figure 7:**
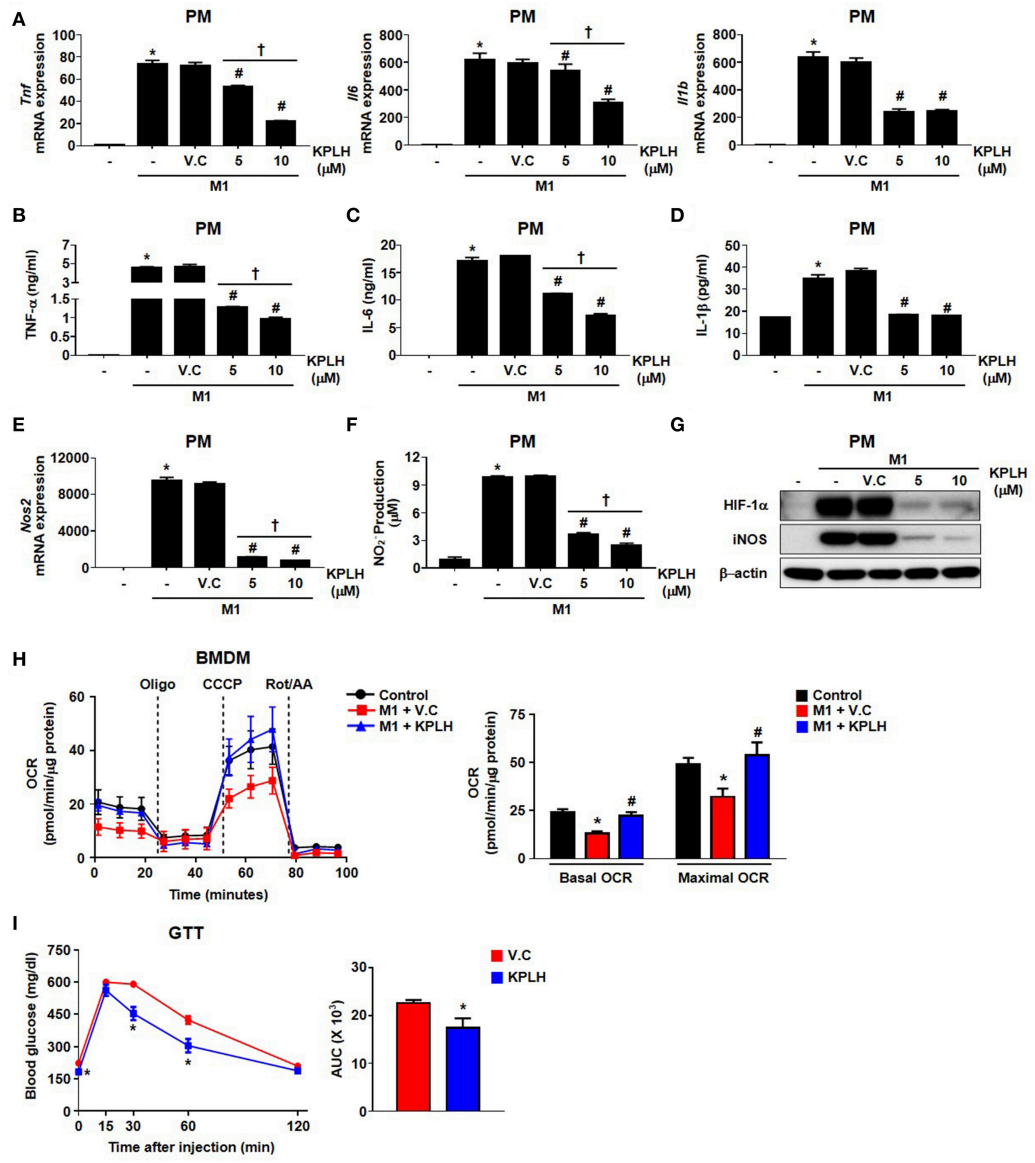
A new PDK inhibitor, KPLH1130, attenuates the secretion of pro-inflammatory effectors by stimulated macrophages and improves glucose tolerance in HFD-fed mice. **(A–F)** The levels of mRNA expression **(A,E)** and secreted proinflammatory effectors **(B–D,F)** were measured with different KPLH1130 concentrations in PMs treated for 12 h with LPS (100 ng/mL) + IFN-γ (10 ng/mL); *n* = 3–6 per group. **(G)** M1 markers were assessed with different KPLH1130 concentrations in PMs treated with LPS (100 ng/mL) + IFN-γ (10 ng/mL) for 12 h. **(H)** OCRs were measured in KPLH1130 (10 μM) ± M1 stimulation by LPS (100 ng/mL) + IFN-γ (10 ng/mL) for 3 h; *n* = 4–5 per group. Values are expressed as mean ± SD. Statistical analysis was performed by Student's *t*-test. ^*^p < 0.05 vs. control, #*p* < 0.05 vs. M1 only, and ^†^*p* < 0.05 vs. KPLH1130 (10 μM). **(I)** Glucose tolerance test (1.5 g/kg, i.p.) after 6 h fasting was measured after HFD for 14 weeks with KPLH1130 (70 mg/kg, 4 weeks); *n* = 6 per group. Values are expressed as mean ± SEM. Statistical analysis was performed by one-way followed by Tukey's HSD. ^*^p < 0.05 vs. vehicle control.

**Figure 8:**
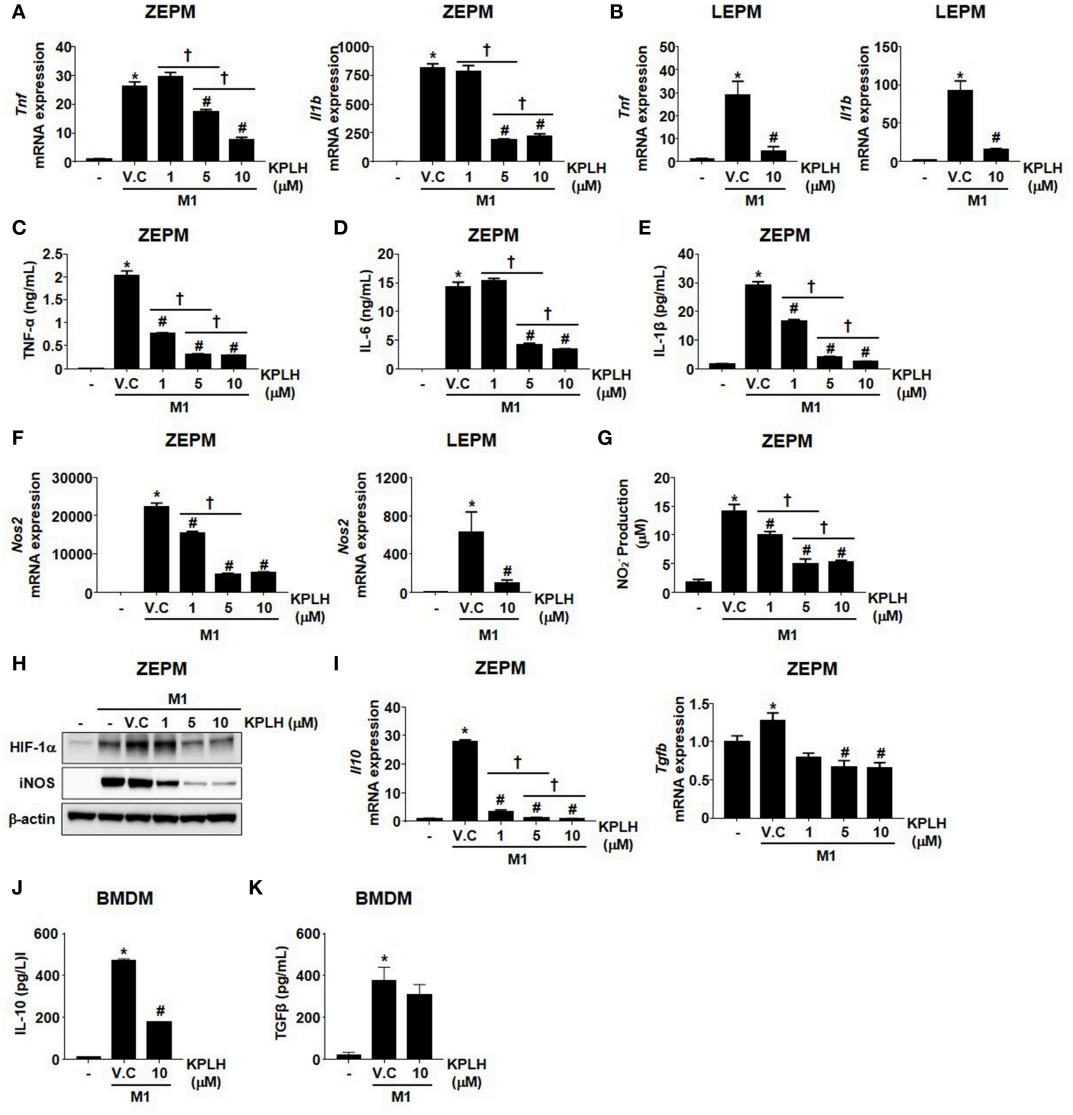
Macrophage activation is suppressed by the novel PDK inhibitor, KPLH1130 using Zymosan-elicited PMs and LPS-elicited PMs. **(A–G)** mRNA expression **(A,B,F)** and secreted proinflammatory effectors **(C–E,G)** were measured following KPLH1130 (10 μM) treatment of ZEPMs or LEPMs incubated with LPS (100 ng/mL) + IFN-γ (10 ng/mL) for 12 h; *n* = 3 per group. **(H)** M1 markers were assessed following KPLH1130 (10 μM) treatment of ZEPMs incubated with LPS (100 ng/mL) + IFN-γ (10 ng/mL) for 12 h. **(I–J)** mRNA expression of IL-10 and TGFβ **(I)** and secreted IL-10 **(J)** and TGFβ **(K)** were measured following KPLH1130 (10 μM) treatment of ZEPMs and BMDMs, respectively incubated with LPS (100 ng/mL) + IFN-γ (10 ng/mL) for 12 h; *n* = 3 per group. Values are expressed as mean ± SD. Statistical analysis was performed using Student's *t*-test. ^*^p < 0.05 vs. control, #*p* < 0.05 vs. M1+V.C and ^†^ *p* < 0.05 vs. different doses of KPLH1130.

We used a series of previously established, highly specific PDK inhibitors that target structurally conserved ATP-binding pockets in the PDKs (23, 24) to perform an efficacy test against the inflammatory response (Figures S5A–C). In contrast to the high DCA concentrations (0.5~2 mM) required for the inhibition of PDKs, a novel PDK inhibitor, KPLH1130, was found to significantly inhibit expression of proinflammatory cytokines including TNFα, IL-6, and IL-1β in various types of macrophages at much lower (5–10 μM) concentrations (Figures 7A–D, 8A–E; Figures S5D–F). In addition to reduced migration capacity by KPLH1130 treatment, iNOS, nitric oxide, and HIF-1α levels were significantly reduced by pharmacological PDK inhibition in various types of macrophages (Figures 7E–G, 8F–H; Figures S5G–M). KPLH1130 also prevented the decrease in basal and maximal OCR caused by M1 polarizing conditions in BMDMs (Figure 7H). We also found that KPLH1130 administration improved the glucose tolerance of HFD-fed mice (Figure 7I). Similarly, these effects were not mediated by induced anti-inflammtory cytokines (Figures 8I–K). Taken together, our results are indicative that KPLH1130 can effectively attenuate inflammatory responses induced by obesity-associated metabolic dysfunction.

## Discussion

This study shows that PDK2/4 deficiency blocks polarization of resting macrophages to the M1 phenotype in response to LPS and INF-γ. Our findings establish that the regulation of PDH activity by the PDKs plays an important role in M1 macrophage polarization. The evidence for this includes a) dramatically induced PDK4 expression by LPS + INF-γ; b) reduced HIF-1α levels and suppression of the Warburg effect (phenomenon characterized by upregulation of glycolytic enzymes and down regulation of TCA cycle enzymes) in response to PDK2/4 deficiency; c) PDK2/4 deficiency-mediated check on the pool size of glycolytic intermediates that would, otherwise, feed into anabolic pathways required for proliferation of macrophages; and d) significantly reduced levels of NO and proinflammatory cytokines including TNFα, IL-6, and IL-1β in response to PDK2/4 deficiency. These findings suggest that sterile inflammatory conditions, such as neuroinflammatory diseases, obesity, and steatohepatitis may be amendable to therapeutic intervention with PDK inhibitors.

Markers of AT inflammation including the number of infiltrated AT macrophages in response to over-nutrition, M1 macrophage population, presence of crown-like structures, and the levels of proinflammatory markers were found to be significantly reduced in the global PDK2/4 DKO mice compared to that in WT mice. These indicators of AT inflammation are associated with the metabolic dysfunctions characteristic of obesity including hyperglycemia, glucose intolerance, and insulin resistance. These findings clearly establish an important role for PDKs and, therefore, the regulation of the activity of the PDH complex in obesity-induced AT inflammation and metabolic dysfunction. To directly determine if these effects could be due to prevention of M1 macrophage polarization by PDK deficiency, macrophage-specific PDK deficiency was induced by transplanting PDK2/4-deficient myeloid cells into irradiated WT mice that were subsequently fed an HFD to induce obesity. Whereas, the AT of obese mice transplanted with normal myeloid cells became inflamed as evidenced by the accumulation of crown-like structures and protein markers for M1 macrophages, the AT of mice transplanted with PDK2/4-deficient cells remained free of inflammation; additionally, the negative effects on blood glucose levels, glucose tolerance, and insulin sensitivity were partially ablated. The observation that glucose tolerance and insulin sensitivity in HFD-fed DKO-BMT mice is only partially improved in contrast to the almost complete protection from HFD-induced glucose intolerance, insulin insensitivity, obesity, and macrophage infiltration observed in the organs of PDK2/4 DKO mice is suggestive that the beneficial effects of PDK deficiency are not totally due to inhibition of macrophage polarization. In other words, increased PDH activity in tissue other than myeloid cells plays a role in the beneficial effects of global PDK2/4 deficiency in HFD-fed mice.

The present study also shows that KPLH1130, a novel PDK-specific inhibitor, blocks M1 polarization and attenuates proinflammatory responses. Additionally, we have observed that M2-polarized PDK2/4 DKO macrophages express elevated levels of arginase-1—a superior competitor to iNOS for the substrate arginine—which has anti-inflammatory functions. The metabolic activation of macrophages triggered by glucose, insulin, and palmitate plays a key role in adipose-driven inflammation which results in insulin resistance—a process distinct from classical activation (25). Both genetic and pharmacological inhibition of PDK in macrophages significantly suppressed proinflammatory responses including M1 polarization markers, bactericidal activity, adherence, and migration. These findings support the notion that PDKs are potential targets for the treatment of inflammation as well as the negative metabolic repercussions of inflammatory conditions.

Gene set enrichment analysis identified the signaling pathways associated with inflammatory responses. Interestingly, the decrease in *Idh* expression, indicating a metabolic break in the TCA cycle of M1-polarized macrophages (26), was significantly attenuated after PDK2/4 deletion. PDK2/4 deletion prevented the reduction in mitochondrial OXPHOS that normally occurs in response to M1 stimulation. A metabolic switch from OXPHOS to aerobic glycolysis is followed by increased production of lactate and the accumulation of citrate and succinate by TLR4 activation (27, 28). According to our stable isotope flux study, PDK 2/4 ablation prevented the metabolic breaks associated with citrate and succinate accumulation as well as the increased lactate production in M1-polarized macrophages. In the case of PDK2 deficiency in hepatocytes, ^13^C-glucose tracer analysis has suggested that PDC flux increases but TCA cycle intermediate flux decreases due to shunted ketogenesis (29).

Although the same effects induced by PDK2/4 deficiency on macrophage polarization are also induced by PDK1 deficiency (9), PDK1 is not sensitive to induction by LPS stimulation (13), and PDK1 deficiency is less effective in preventing the expression of the glycolytic enzymes (9). Nevertheless, the finding that deficiencies of PDK1 and PDK2/4 block macrophage polarization to the proinflammatory phenotype indicates that reducing PDH activity by phosphorylation is required for LPS-induced macrophage polarization. Surprisingly, it has been reported that LPS-induced macrophage polarization is also prevented by a number of manipulations that strongly inhibit PDH flux. These include knockdown of pyruvate dehydrogenase phosphatase 1 (12), pharmacological inhibition of pyruvate import into the mitochondria (13), and upregulation of PDK2 by VSIG4 (14). These findings suggest that complete inhibition of PDH is not compatible with proinflammatory macrophage polarization. Indeed, pyruvate oxidation through PDH is necessary for the synthesis of the antimicrobial metabolite itaconate, an important product of mature proinflammatory macrophages (13). The finding that macrophage polarization is prevented by both inhibition of PDH and PDK deficiency suggests that LPS-induced polarization of macrophages is extremely sensitive to PDH flux. This is consistent with the evidence that reprogramming of the mitochondrial processes, particularly glutaminolysis, is also necessary for the inflammatory responses of M1 macrophages (30). If flux through PDH is too high or too low, changes in gene expression, reprogramming of glycolysis and the TCA cycle, and polarization to the M1 phenotype are prevented. Perhaps changes in the levels of metabolites transmit information to the nucleus when conditions are right for the reprogramming of gene expression necessary for polarization. The location of PDH allows it to precisely control the relationship between glycolysis and the TCA cycle and therefore serves as a sensitive checkpoint of bioenergetic reprogramming. In spite of the complexity, this makes PDH a sensitive target for therapeutic intervention in inflammatory conditions, suggesting that PDK2 and PDK4 in immune cells are potential targets for the treatment of inflammatory metabolic disorders.

## Ethics Statement

All experiments were approved by the Institutional Animal Care and Use Committee of Kyungpook National University and were conducted according to recommendations in the National Institutes of Health Guide for the Care and Use of Laboratory Animals.

## Author Contributions

B-KM, SP, DK, RAH, and I-KL conceived and designed research. B-KM, H-JK, HH, C-MH, B-JC, JYL, CO, EY, HK, and DYH performed experiments. B-KM, SP, B-GK, DH, Y-HK, TL, and C-HL analyzed data. B-KM, SP, J-HJ, RAH, and I-KL interpreted results of experiments. B-KM and SP prepared figures. B-KM, SP, RAH, and I-KL drafted manuscript. B-KM, SP, JK, Y-KC, K-GP, AC, JL, RAH, and I-KL edited and revised the manuscript. B-KM, SP, H-JK, RAH, and I-KL approved final version of manuscript.

### Conflict of Interest Statement

The authors declare that the research was conducted in the absence of any commercial or financial relationships that could be construed as a potential conflict of interest.
