# Supplementary material for: Pyruvate Dehydrogenase Kinase Is a Metabolic Checkpoint for Polarization of Macrophages to the M1 Phenotype
Source: Front Immunol. 2019 May 7;10:944. doi: 10.3389/fimmu.2019.00944 (PMC6514528; doi:10.3389/fimmu.2019.00944)
Supplement: Supplementary file 1 [file Data_Sheet_1.pdf]

Supplemental Data

Pyruvate dehydrogenase kinase is a metabolic checkpoint for polarization of macrophages to the M1 phenotype

Byong-Keol Min[1,3†], Sungmi Park[2†], Hyeon-Ji Kang[3†], Dong Wook Kim[2], Hye Jin Ham[2], Chae-Myeong Ha[1], Byung-Jun Choi[1], Jung Yi Lee[2], Chang Joo Oh[3], Eun Kyung Yoo[2], Hui Eon Kim[2], Byung-Gyu Kim[2], Jae-Han Jeon[4], Do Young Hyeon[5], Daehee Hwang[5,6], Yong-Hoon Kim[7], Chul-Ho Lee[7], Taeho Lee[8], Jung-whan Kim[9], Yeon-Kyung Choi[4], Keun-Gyu Park[4], Ajay Chawla[10], Jongsoon Lee[11], Robert A. Harris[12*], and In-Kyu Lee[1,2,3,4*]

[1]Department of Biomedical Science & BK21 plus KNU Biomedical Convergence Programs, Kyungpook National University, Daegu, South Korea

[2]Leading-edge Research Center for Drug Discovery and Development for Diabetes and Metabolic Disease, Kyungpook National University Hospital, Daegu, South Korea

[3]Research Institute of Aging and Metabolism, Kyungpook National University, Daegu, South Korea

[4]Department of Internal Medicine, School of Medicine, Kyungpook National University, Kyungpook National University Hospital, Daegu, South Korea

[5]Department of Biological Sciences, Seoul National University, Seoul, Republic of Korea

[6]Center for Plant Aging Research, Institute for Basic Science, Daegu Gyeongbuk Institute of Science and Technology, Daegu, Republic of Korea

[7]Laboratory Animal Resource Center, Korea Research Institute of Bioscience and Biotechnology, Daejeon, South Korea

[8]College of Pharmacy, Kyungpook National University, Daegu, South Korea

[9]Department of Biological Sciences, The University of Texas at Dallas, Richardson, TX, USA

[10]Department of Medicine, University of California, San Francisco, CA, USA

[11]Soonchunhyang Institute of Medi-bio Science, Soon Chun Hyang University, Cheonan, South Korea

[12]Department of Biochemistry and Molecular Biology, Indiana University School of Medicine, Indianapolis, IN, USA

[†]These authors contributed equally to this work.

[*]Correspondences:

In-Kyu Lee, M.D., Ph.D.

leei@knu.ac.kr

Robert A. Harris, Ph.D.

raharris@iu.edu

Supplemental Material

L929 conditioned media

L929 conditioned media was prepared as previously reported(Weischenfeldt and Porse, 2008). Briefly, 4.7 × 10^5 L929 cells were seeded in a 75 cm^2 flask in 55 mL of DMEM-high glucose (WELGENE; LM 001-05) containing 10% FBS, 10 mM HEPES, 1X GlutaMax-I (Gibco; 35050), and antibiotics. Cells were cultured for 7 days in a humidified incubator with 5% CO_2 at 37 °C. Culture supernatant was collected and filtered using a 0.2-µm filter and then stored at –80 °C.

Nitric oxide (NO) secretion level

Secreted levels of NO in culture supernatants were measured using the Griess reagent system (Promega; TB229) following the manufacturer's instructions.

Bactericidal efficacy test

Bactericidal activities of WT- or DKO-PMs were determined as previously reported(Liao et al., 2011). Briefly, 5 × 10^6 PMs were incubated with 2.5 × 10^8 *E. coli* for 2 h at 37 °C. The cells were then thoroughly washed with PM culture media containing antibiotics followed by a wash with PBS, then incubated for 24 h at 37 °C in PM culture media containing antibiotics. The cells were then collected by scraping in deionized water and plated onto agar plates to determine bacterial viability.

Cell adhesion assay

Cell adhesion assay was performed as previously reported(Min et al., 2013). Briefly, 96-well plates were coated with 10 µg/mL of collagen and incubated overnight on a clean bench under UV light. Six-well plates were seeded with 1 × 10^6 THP-1 cells, and serum starvation was performed for 6 h. The cells were then treated with DCA and incubated overnight. Thereafter, 5 × 10^4 cells were transferred to collagen-coated wells in serum-free media in the presence or absence of DCA and LPS. After 24 h, unbound cells were eliminated by washing with cold PBS followed by triplicate washes using serum-free media. The Cell Counting Kit-8 (CCK-8) solution (Dojindo; CK04) was added to each well and incubated for 1 h. Absorbance at 450 nm was measured by a microplate reader (Molecular Devices; VERSA max).

3T3-L1 conditioned media

3T3-L1 cells were differentiated as previously reported(Kang et al., 2013). After 8 days, the cells were washed before being treated with BSA or BSA-conjugated palmitate (500 µM) for 24 h. After the treatments, the media was collected from the incubated cells.

Isolation of the stromal vascular fraction (SVF) from the epididymal AT

Epididymal AT was dissected and digested with type I collagenase (Worthington; CLS-1) for 20 min at 37 °C. An equal volume of α-MEM medium containing 10% FBS was added to stop the reaction. After filtration through a 100-µm mesh filter, the media was centrifuged at 1500 rpm for 5 min and the cell pellet resuspended in α-MEM medium containing 10% FBS. The resulting cell suspension was filtered through a 40-µm mesh filter and then centrifuged at 1500 rpm for 5 min. The cell pellet was resuspended in FACS buffer for FACS analysis using BD Accuri™ C6 flow cytometer (BD Biosciences, CA, USA).

In vivo migration

In vivo migration assays were performed as described previously (Oh et al., 2012). Briefly, monocytes were isolated from 8-week-old mice and enriched using an EasySep mouse monocyte enrichment kit (STEMCELL Technologies; 19761) according to the manufacturer's instructions. Isolated monocytes were stained with PKH26 (Sigma Chemical; PKH26GL) at 5 mM, and 1 × 10^6 stained, viable cells were injected into the tail vein of 4 week HFD-fed recipient mice. Two days after the injection, the macrophages were isolated from epididymal AT and the PKH26-positive macrophages (PKH26+F4/80+CD11b+) were analyzed by BD AccuriTM C6 flow cytometer (BD Biosciences, CA, USA).

Flow cytometry analysis

Isolated SVFs from the epididymal AT were incubated with Fc blocker (BD Biosciences; 553142) for 5 min on ice. The cells were then incubated with F4/80 (eBioscience, #45-4801-82), CD11c (eBioscience; 12-0114-82), CD11b (BD Biosciences; 561039), and CD206 (BioLegend; 141703) for 1 h on ice. The size of the cell population and protein levels were determined using the BD Accuri™ C6 cytometer. The acquired data was analyzed by FlowJo (version X) flow cytometry analysis software (FlowJo, LLC).

FACS analysis for LPS-induced PM polarization in vivo

LPS-activated PMs were isolated as previously described(Ni Gabhann et al., 2014). Briefly, WT or PDK2/4 DKO mice were i.p. injected with LPS (1 mg/kg). Twenty-four hours later, the mice were sacrificed, and the peritoneal fluids were isolated using the standard PM isolation method. Isolated peritoneal fluid was incubated with Fc blocker (BD Biosciences; 553142) for 5 min on ice. The cells were then incubated with antibodies against (eBioscience; 45-4801-82), CD11c (eBioscience; 12-0114-82), CD11b (BD Biosciences; 561039), and CD206 (BioLegend; 141703) for 1 h on ice. Cell populations were analyzed by FACS analysis.

Enzyme-linked immunosorbent assay (ELISA) for cytokines

Secreted levels of TNF-α, IL-6, IL-1β, MCP-1, TGFβ, and IL-10 were determined in cell culture media using ELISA Ready Set-Go kits (eBioscience) or R&D system according to the manufacturer's instructions, respectively.

Glucose & insulin tolerance test (GTT & ITT)

GTT was performed by injecting mice with D-glucose (1.5 g/kg) i.p. after 16 h fasting. Blood glucose levels were measured at the indicated time points with a standard glucometer (Roche; Accu-Check Active). Likewise, for ITT, insulin (0.75 U/kg) was injected i.p. into mice after a 6 h period of fasting; blood glucose levels were measured at indicated time points using a glucometer.

LC-MS/MS analysis

The samples were separated on a pentafluorophenyl column (100 × 2.1 mm, 3 μm) by gradient elution using an HPLC Nexera coupled with an LCMS-8060 mass spectrometer (Shimadzu, Japan). The mobile phase consisted of water-acetonitrile in 0.1% formic acid at a flow rate of 0.2 mL/min. The Q3 selected ion monitoring (SIM) scan mode was used to obtain target metabolite isotopomer information, and raw spectrum intensity data were extracted from within a retention time range for each multiple reaction monitoring scan

performed simultaneously. Subsequently, ^{13}C-mass isotopomer distributions were determined and corrected for natural isotope abundance from the SIM scan data for target metabolite isotopomers.

Quantitative real-time polymerase chain reaction (qPCR)

Total RNA was extracted from mouse tissue or cells by QIAzol lysis reagent (Qiagen; 79306) following the manufacturer's instructions. Total RNA (2 μg) was used for cDNA synthesis by the cDNA synthesis kit (Thermo Fisher Scientific; K1621). The qPCR analysis was performed with SYBR Green (Thermo Fisher Scientific; 4367659) using the ViiA 7 real-time PCR system (Applied Biosystems, USA). 36B4 was used as an endogenous control.

mRNA-sequencing and data analysis

PMs were isolated from 8- to 10-week-old WT and DKO mice. For mRNA-sequencing, total RNAs were prepared from WT PMs untreated (Cont-WT) or treated with LPS (100 ng/mL) and IFN-γ (10 ng/mL) for 12 h (M1-WT), and DKO PMs treated with LPS and IFN-γ for 12 h (M1-DKO). Total RNAs were purified with QIAzol lysis reagent (QIAGEN; 79306) and RNeasy Plus Micro kit (QIAGEN; 74034). Poly(A) mRNA isolation from total RNAs and fragmentation were performed using the Illumina Truseq Stranded mRNA LT Sample Prep Kit with poly-T oligo-attached magnetic beads, according to the manufacturer's protocol. The adaptor ligated libraries were sequenced using an Illumina Hi-Seq 2500 (Illumina, USA). From the resulting read sequences for each sample, adapter sequences (TruSeq universal and indexed adapters) were removed using the cutadapt software (ver. 1.4.2)(Martin, 2011). The resulting reads were then aligned to the *Mus musculus* reference genome (GRCm38) using TopHat aligner (ver. 2.0.10)(Trapnell et al., 2009) with the default options. After the alignment, we counted the mapped reads for gene features (GTF file of GRCm38) using HTSeq (ver. 0.6.1)(Anders et al., 2015) and also estimated fragments per kilobase of transcript per million fragments mapped (FPKM) using Cufflinks (ver. 2.1.1)(Trapnell et al., 2010). The raw and normalized data of mRNA-sequencing were deposited at the Gene Expression Omnibus database (GSE112595).

Identification of differentially expressed genes (DEGs)

We first identified 'expressed' genes as the ones with FPKM larger than 1 in at least one of the six samples (two samples per condition). For these expressed genes, the number of reads counted by HTseq were normalized using the TMM normalization method(Robinson and Oshlack, 2010) provided in the edgeR package(Robinson et al., 2010). The normalized counts were converted to $\log_2$-read counts after adding one to the normalized counts. The $\log_2$-read counts for the samples were then normalized using the quantile normalization method(Bolstad et al., 2003). For each gene, we calculated T-statistic values using Student's *t*-test and also $\log_2$-fold-changes in the two comparisons: M1-WT versus Cont-WT (M1-WT/Cont-WT) and M1-DKO versus M1-WT (M1-DKO/M1-WT). We then estimated empirical distributions of T-statistics and $\log_2$-fold-change for the null hypothesis by random permutation of the six samples. Using the estimated empirical distributions, we computed adjusted *p*-values for the two tests for each gene and then combined these *p*-values with Stouffer's method(Hwang et al., 2005). Finally, we identified DEGs as the ones that have the combined p-values < 0.05 and absolute $\log_2$-fold-changes > 0.58 (1.5-fold). To identify cellular processes represented by the DEGs, we performed the enrichment analysis of gene ontology biological processes (GOBPs) for the genes using DAVID software(Huang et al., 2009) and selected the GOBPs with p-value < 0.05 as the processes enriched by the DEGs.

Reagents

REAGENT or RESOURCE	SOURCE	IDENTIFIER
Antibodies		
Anti-PDK2 antibody	Santa Cruz Biotechnology	sc-100534
Anti-PDK4 antibody	(Jeoung and Harris, 2008)	N/A
Anti-iNOS antibody	BD Biosciences	610328
Anti-HIF-1α antibody	Novus Biologicals	NB100-479
Anti-Arg-1 antibody	Santa Cruz Biotechnology	sc-18351
Anti-F4/80 antibody	Thermo Fisher Scientific	MA1-91124
Anti-β-actin antibody	Sigma-Aldrich	A5441
Anti-F4/80-PecCP-Cy5.5 antibody	eBioscience	45-4801-82
Anti-CD11b-APC-Cy7 antibody	BD Biosciences	561039
Anti-CD11c-PE antibody	eBioscience	12-0114-82
Anti-CD206-FITC antibody	BioLegend	141703
Anti-IgG2a-PecCP-Cy5.5 antibody	eBioscience	45-4321-80
Anti-IgG2b-APC-Cy7 antibody	BD Biosciences	552773
Anti-IgG-PE antibody	eBioscience	12-4888-81
Anti-IgG2a-FITC antibody	BioLegend	400505
Anti-CD16/CD32 antibody (Fc blocker)	BD Biosciences	553142
Chemicals, Recombinant Proteins, and Analysis kits		
Thioglycollate	BD Biosciences	211716
LPS	Sigma-Aldrich	L4391
Zymosan A	Sigma-Aldrich	Z4250
Recombinant rat IFN-γ	R&D Systems	585-IF-100
Recombinant mouse IL-4	R&D Systems	404-ML-010
Recombinant human IL-10	R&D Systems	217-IL-005
Recombinant mouse CCL2 (MCP-1)	R&D Systems	479-JE-010
Oligomycin A	Sigma-Aldrich	75351
CCCP	Sigma-Aldrich	C2759
Rotenone	Sigma-Aldrich	R8875
Antimycin A	Sigma-Aldrich	A8674
D-Glucose-$^{13}C_6$	Sigma-Aldrich	389374
Collagenase, Type 1	Worthington	CLS-1
Collagen, Bovine, Type 1	BD Biosciences	354231
Sodium dichloroacetate (DCA)	Sigma-Aldrich	347795
Aprotinin	AMRESCO	E429
Leupeptin	AMRESCO	M180
QIAzol lysis reagent	Qiagen	79306
Power SYBR™ Green PCR Master Mix	Thermo Fisher Scientific	4367659
cDNA synthesis kit	Thermo Fisher Scientific	K1621
Phosphatase inhibitor cocktail 3	Sigma-Aldrich	P0044
Succinate assay kit	Abnova	KA3955
RNeasy Plus Micro kit	Qiagen	74034
Mouse TNF-α ELISA	Thermo Fisher Scientific	88-7324
Mouse IL-6 ELISA	Thermo Fisher Scientific	39-8061
Mouse IL-1β ELISA	Thermo Fisher Scientific	29-8012
Mouse IL-10 ELISA	R&D Systems	M1000B
Mouse TGFβ ELISA	Thermo Fisher Scientific	88-8350
Human TNF-α ELISA	Thermo Fisher Scientific	88-7346

Human IL-8 ELISA	Thermo Fisher Scientific	29-8089
Human IL-1β ELISA	Thermo Fisher Scientific	29-8018
Human MCP-1 ELISA	Thermo Fisher Scientific	88-7399
Griess reagent system	Promega	TB229
Mouse monocyte enrichment kit	STEMCELL Technologies	19761
PKH26 cell linker kit	Sigma-Aldrich	PKH26GL
Critical Commercial Assays		
Truseq Stranded mRNA LT Sample Prep Kit	Illumina	RS-122-2101
Software and Algorithms		
cutadapt v1.4.2	(Martin, 2011)	http://cutadapt.readthedocs.io/en/stable/index.html
TopHat v2.0.10	(Trapnell et al., 2009)	https://ccb.jhu.edu/software/tophat/index.shtml
Bowtie v2.1.0.0	(Langmead and Salzberg, 2012)	http://bowtie-bio.sourceforge.net/bowtie2/index.shtml
Samtools v0.1.18.0	(Li et al., 2009)	http://samtools.sourceforge.net/
Picard v1.85		https://broadinstitute.github.io/picard/
HTSeq v0.6.1	(Anders et al., 2015)	http://www-huber.embl.de/HTSeq/doc/overview.html
Cufflinks v2.1.1	(Trapnell et al., 2010)	http://cole-trapnell-lab.github.io/cufflinks/
edgeR R package	(Robinson et al., 2010)	https://bioconductor.org/packages/release/bioc/html/edgeR.html
DAVID v6.8	(Huang et al., 2009)	https://david.ncifcrf.gov/home.jsp
Deposited Data		
Raw and analyzed data	this paper	GEO: GSE112595
ENSEMBL GRCm38	(Yates et al., 2016)	http://www.ensembl.org/index.html

Quantitative real-time PCR primers

Gene name (mouse)	Sequence
Pdk1 - Forward	CACCACGCGGACAAAGG
Pdk1 - Reverse	GCCCAGCGTGACGTGAA
Pdk2 - Forward	CCCCGTCCCCGTTGTC
Pdk2 - Reverse	TCGCAGGCATTGCTGGAT
Pdk3 - Forward	GGAGCAATCCCAGCAGTGAA
Pdk3 - Reverse	TGATCTTGTCCTGTTTAGCC
Pdk4 - Forward	CCATGAGAAGAGCCCAGAAGA
Pdk4 - Reverse	GAACTTTGACCAGCGTGTCTACAA
Emr1 (F4/80) - Forward	TGGCTGCCTCCCTGACTTT
Emr1 (F4/80) - Reverse	TCCTTTTGCAGTTGAAGTTTCCAT
Itgax (CD11c) - Forward	CTGGGCCTGTCCCTTGCT
Itgax (CD11c) - Reverse	ACAGTAGGACCACAAGCGAACA
CD68 - Forward	CGCTTATAGCCCAAGGAACA
CD68 - Reverse	CGTAGGGCTGGCTGTGCTT
TNF-α - Forward	GACGTGGAACTGGCAGAAGA
TNF-α - Reverse	CCGCCTGGAGTTCTGGAA
Ccl2 (Mcp-1) - Forward	CCCACTCACCTGCTGCTACTCA
Ccl2 (Mcp-1) - Reverse	GCTTCTTTGGGACACCTGCTG
IL-6 - Forward	GTTGCCTTCTTGGGACTGAT
IL-6 - Reverse	TTGGGAGTGGTATCCTCTGT
IL-1β - Forward	GAGCACCTTCTTTTCCTTCATCTT
IL-1β - Reverse	TCACACACCAGCAGGTTATCATC
Nos2 (iNOS) - Forward	GGCAGCCTGTGAGACCTTTG
Nos2 (iNOS) - Reverse	TGCATTGGAAGTGAAGCGTTT
IL-10 - Forward	CCAAGCCTTATCGGAAATGA
IL-10 - Reverse	TTTTCACAGGGGAGAAATCG
TGFβ - Forward	TGCTAATGGTGGACCGCAA
TGFβ - Reverse	CACTGCTTCCCGAATGTCTG
36B4 - Forward	ACCTCCTTCTTCCAGGCTTT
36B4 - Reverse	CTCCAGTCTTTATCAGCTGC

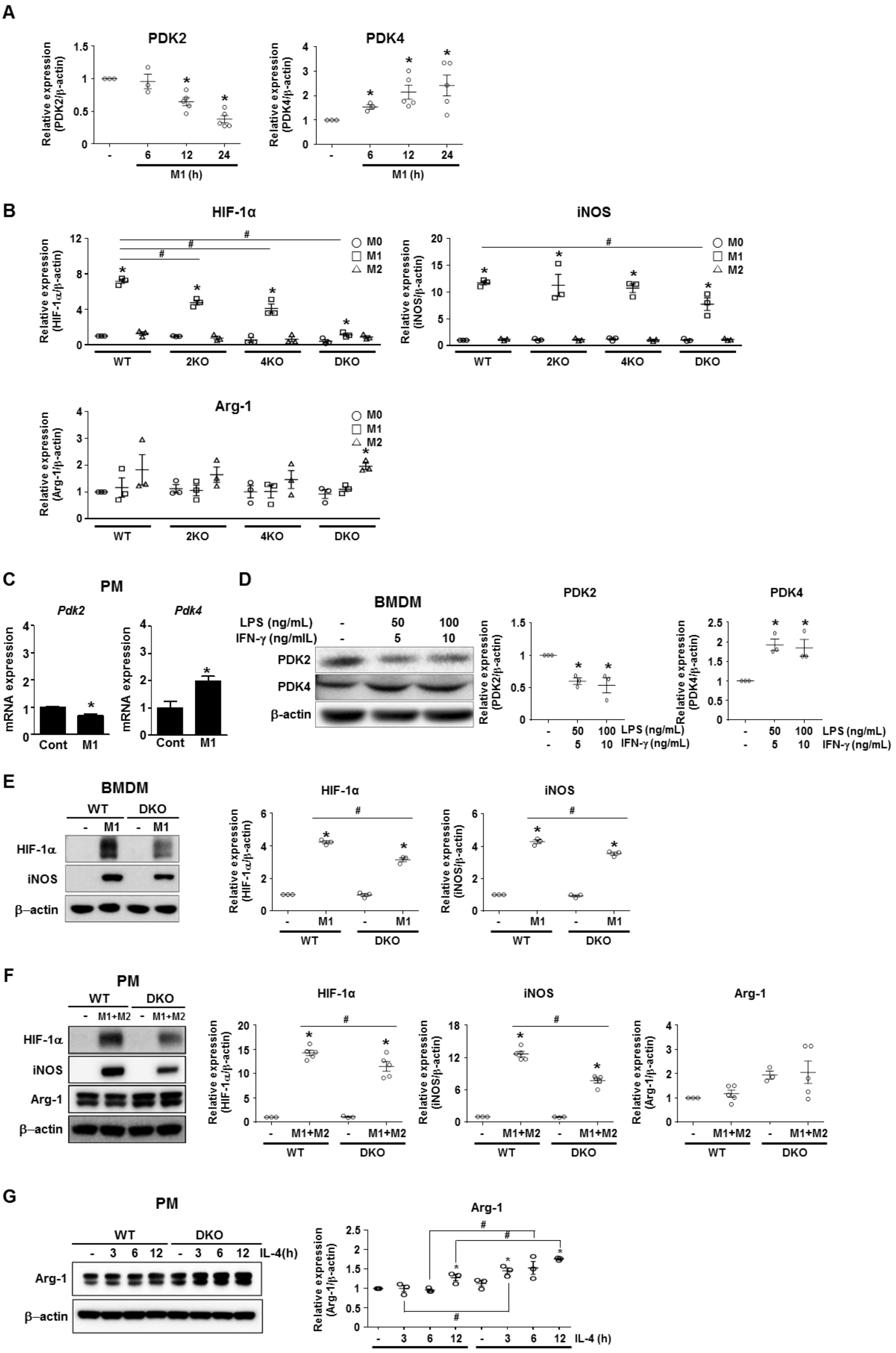
A
PDK2
Relative expression (PDK2/β-actin)
1.5
1
0.5
0
-
6
12
24
M1 (h)
PDK4
Relative expression (PDK4/β-actin)
4
3
2
1
0
B
HIF-1α
Relative expression (HIF-1α/β-actin)
12
8
4
0
#
M0
M1
M2
WT
2KO
4KO
DKO
iNOS
Relative expression (iNOS/β-actin)
20
15
10
5
Arg-1
Relative expression (Arg-1/β-actin)
C
PM
Pdk2
Pdk4
mRNA expression
Cont
M1
D
BMDM
LPS (ng/mL)
IFN-γ (ng/mL)
50
100
5
10
PDK2
PDK4
β-actin
LPS (ng/mL)
IFN-γ (ng/mL)
Relative expression (PDK2/β-actin)
Relative expression (PDK4/β-actin)
2.5
E
BMDM
WT
DKO
HIF-1α
iNOS
β–actin
Relative expression (HIF-1α/β-actin)
Relative expression (iNOS/β-actin)
6
F
PM
M1+M2
Arg-1
Relative expression (Arg-1/β-actin)
18
G
PM
IL-4(h)
IL-4 (h)
Arg-1
β–actin
Relative expression (Arg-1/β-actin)

Figure S1. Expression levels of PDK2 and PDK4 in M1 and M2 macrophages. (A) Densitometric analysis graphs for the individual western blotting results related with Fig. 1B. (B) Densitometric analysis graphs for the individual western blotting results related with Fig. 1C. (C) mRNA expressions levels of PDK isoforms were measured in PMs after LPS (100 ng/mL) + IFN-γ (10 ng/mL) stimulation for 24 h; n = 3 per group. Values are expressed as mean ± standard deviation (SD). Statistical analysis was performed using Student's *t* test. *p < 0.05 vs. control, #p < 0.05 vs. M1 or M2 activation. (D) PDK expression levels in BMDMs after M1 stimulation with different LPS + IFN-γ concentrations. (E) Arg-1 protein levels were assessed in PMs after stimulation with IL-4 (5 ng/mL) at the indicated times. (F-G) M1 and M2 markers in BMDMs and PMs prepared from WT- and DKO mice after treatment with or without a combination of M1 stimulants [LPS 100 ng/mL+ IFN- γ 10 ng/mL] and M2 stimulants [IL-4 5 ng/mL], respectively.

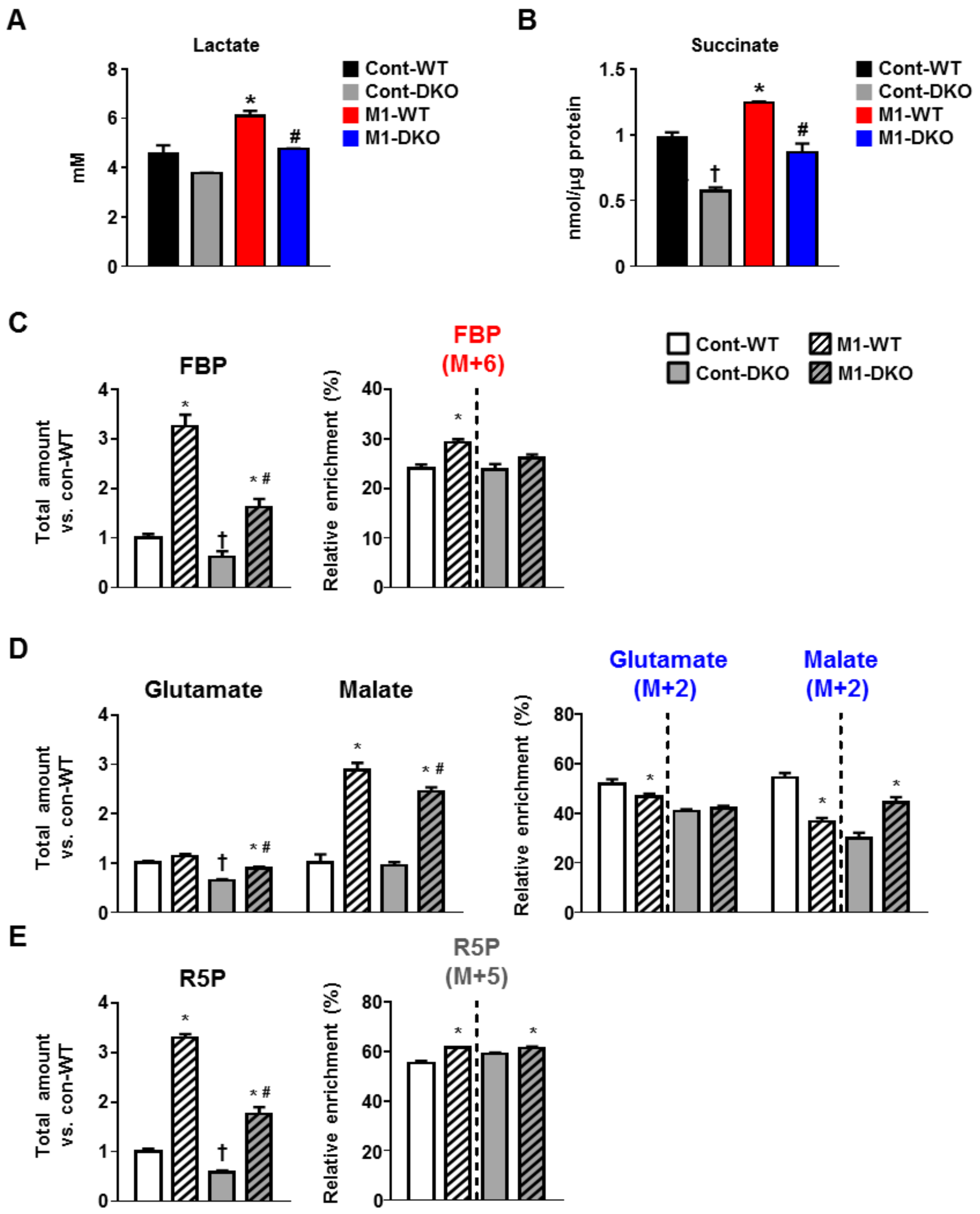

Figure S2. Deficiency of PDK2 and PDK4 drives dynamic conversion of the intermediates in glycolysis and TCA cycle. (A and B) Extracellular lactate levels (A) and intracellular succinate levels (B) after LPS (100 ng/mL) + IFN- γ (10 ng/mL) stimulation of PMs for 12 h; n = 3 per group. Values are expressed as mean ± SD. Statistical analysis was performed using Student's *t* test. *p < 0.05 vs. (-) M1 stimulation and #p < 0.05 vs. M1-WT. (C–E) The relative amounts and ^{13}C enrichment patterns of the intermediates of the glycolytic cycle (C), TCA cycle (D), and pentose phosphate pathway (E) in WT- and DKO-PM compared with control-WT; n = 4–5 per group. Values are expressed as mean ± SEM. Statistical analysis was performed using Student's *t* test. *p < 0.05 vs. (-) M1 stimulation, †p < 0.05 vs. control-WT, and #p < 0.05 vs. M1-WT.

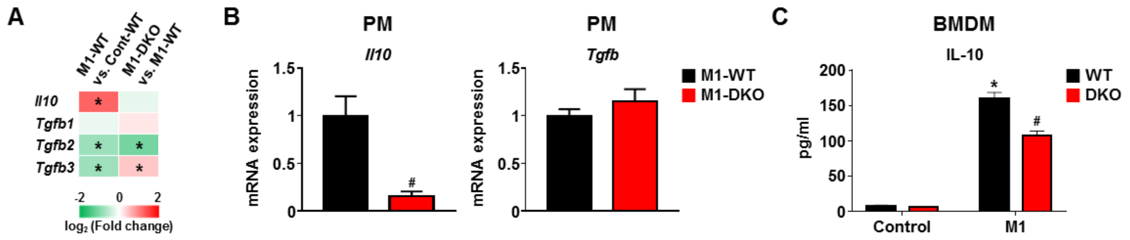

Figure S3. The beneficial effect of PDK2/4 deficiency is not mediated by the induction of anti-inflammatory responses. (A) Heat map of genes involved in anti-inflammatory cytokines like IL-10 and Tgfb1/2/3 by RNA-sequencing analysis. The color bar represents the gradient of log2 (fold change) of mRNA expression levels in each comparison. The genes that were up- (down-) regulated in M1-WT compared to Control-WT and down- (up-) regulated in M1-DKO compared to M1-WT at the same time were indicated by an asterisk. (B) mRNA expressions of IL-10 and Tgfb. (C) Secreted IL-10 level. Values are expressed as mean ± SD Statistical analysis was performed by Student's t test. *p < 0.05 vs. Control-WT, and #p < 0.05 vs. M1-WT.

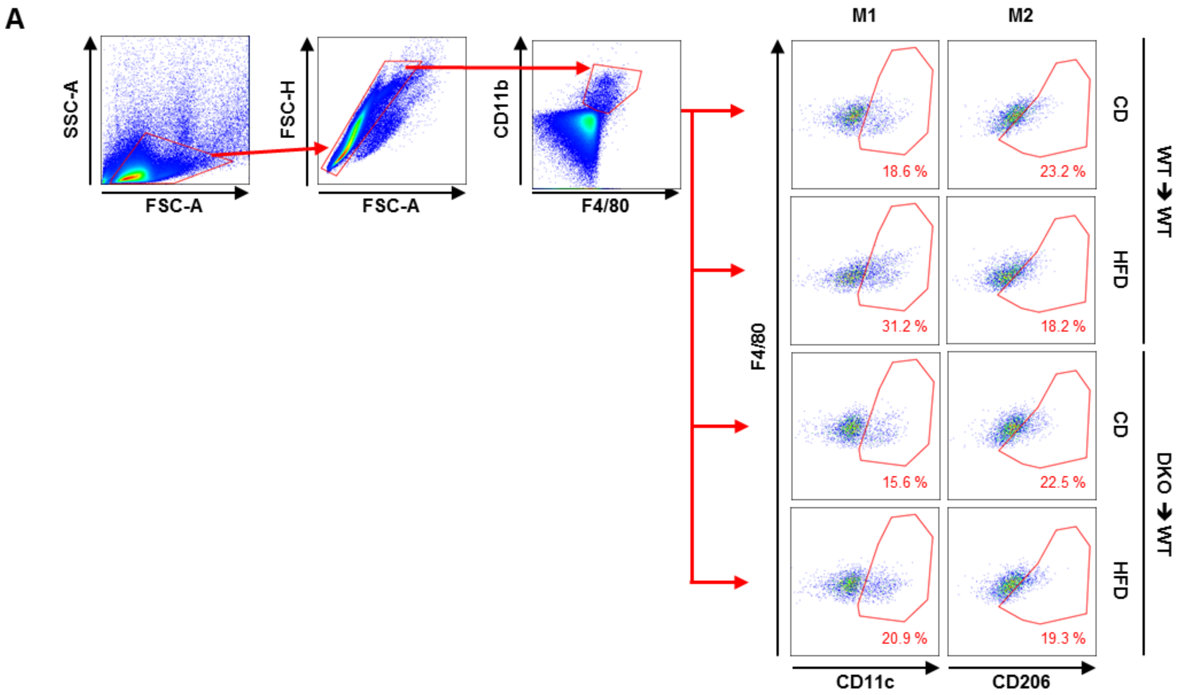

Figure S4. Hematopoietic cell-specific PDK2/4 deletion alters the epididymal AT macrophage polarization. Representative flow cytometric profiles of M1 and M2 macrophage populations in the SVF of the epididymal AT from WT- and DKO-BMT mice. SVF, stromal vascular fraction.

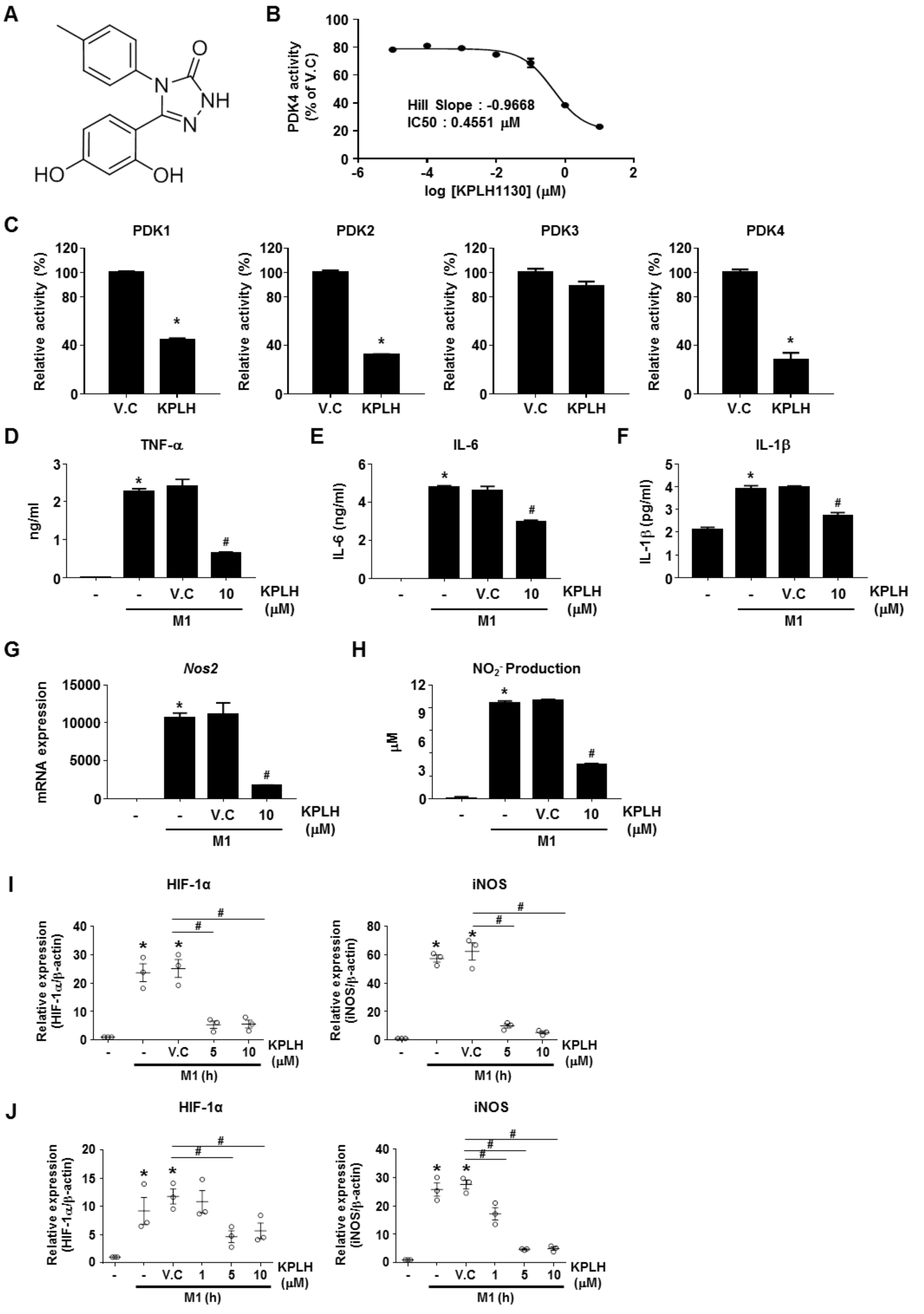
A
O
N
NH
N
HO
OH
B
PDK4 activity
(% of V.C)
Hill Slope : -0.9668
IC50 : 0.4551 μM
log [KPLH1130] (μM)
C
PDK1
PDK2
PDK3
PDK4
Relative activity (%)
V.C
KPLH
D
TNF-α
ng/ml
E
IL-6
IL-6 (ng/ml)
F
IL-1β
IL-1β (pg/ml)
KPLH
(μM)
M1
G
Nos2
mRNA expression
H
NO2- Production
μM
I
HIF-1α
Relative expression
(HIF-1α/β-actin)
iNOS
Relative expression
(iNOS/β-actin)
M1 (h)
J
HIF-1α
iNOS

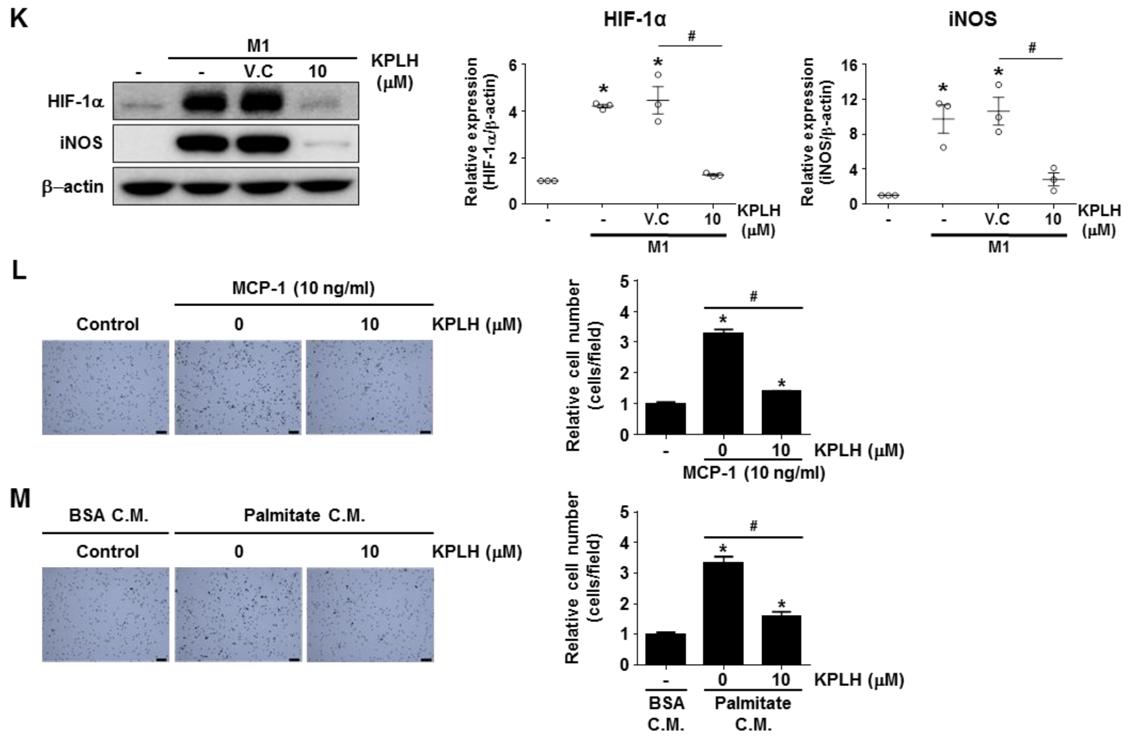

Figure S5. Macrophage activation is suppressed by the novel PDK inhibitor, KPLH1130. (A) Molecular structure of KPLH1130. (B) IC50 value of KPLH1130 determined by measurement of PDK4 activity. (C) Inhibition of PDK isoform activity by KPLH1130 measured by in vitro assay; n = 4 per group. Values are expressed as mean ± SEM. Statistical analysis was performed using Student's *t* test. *p < 0.05 vs. V.C. (D–H) Secreted proinflammatory effectors (D, E, F, and H) and mRNA expression (G) were measured following KPLH1130 (10 μM) treatment of BMDMs incubated with LPS (100 ng/mL) + IFN-γ (10 ng/mL) for 12 h; n = 3–4 per group. (I) Densitometric analysis graphs for the individual western blotting results related with Fig. 7G. (J) Densitometric analysis graphs for the individual western blotting results related with Fig. 8H. (K) M1 markers were assessed following KPLH1130 (10 μM) treatment of PMs incubated with LPS (100 ng/mL) + IFN-γ (10 ng/mL) for 12 h. Statistical analysis was performed by Student's t test. *p < 0.05 vs. control, #p < 0.05 vs. vehicle in M1 activation. (L) MCP-1-induced migration was evaluated in KPLH1130-treated BMDMs using a trans-well migration assay; n = 4 per group; magnification: 200X; scale bar: 50 μm. The relative number of migrated cells per field was counted. Values are expressed as mean ± SEM. Statistical analysis was performed by Student's *t* test. *p < 0.05 vs. control, #p < 0.05 vs. MCP-1 induction. (M) 3T3-L1-conditioned medium (C.M.)-induced migration was evaluated in KPLH1130-treated BMDMs using a trans-well migration assay; n = 4 per group; magnification: 200X; scale bar: 50 μm. The relative number of migrated cells per field was counted. Values are expressed as mean ± SEM. Statistical analysis was performed by Student's *t* test. *p < 0.05 vs. control, #p < 0.05 vs. palmitate C.M.

References

Anders, S., Pyl, P.T., and Huber, W. (2015). HTSeq--a Python framework to work with high-throughput sequencing data. *Bioinformatics* 31**,** 166-169.

Bolstad, B.M., Irizarry, R.A., Astrand, M., and Speed, T.P. (2003). A comparison of normalization methods for high density oligonucleotide array data based on variance and bias. *Bioinformatics* 19**,** 185-193.

Huang, D.W., Sherman, B.T., and Lempicki, R.A. (2009). Systematic and integrative analysis of large gene lists using DAVID bioinformatics resources. *Nature Protocols* 4**,** 44-57.

Hwang, D., Rust, A.G., Ramsey, S., Smith, J.J., Leslie, D.M., Weston, A.D., De Atauri, P., Aitchison, J.D., Hood, L., Siegel, A.F., and Bolouri, H. (2005). A data integration methodology for systems biology. *Proc Natl Acad Sci U S A* 102**,** 17296-17301.

Jeoung, N.H., and Harris, R.A. (2008). Pyruvate dehydrogenase kinase-4 deficiency lowers blood glucose and improves glucose tolerance in diet-induced obese mice. *American journal of physiology. Endocrinology and metabolism* 295**,** E46-54.

Kang, H.J., Seo, H.A., Go, Y., Oh, C.J., Jeoung, N.H., Park, K.G., and Lee, I.K. (2013). Dimethylfumarate suppresses adipogenic differentiation in 3T3-L1 preadipocytes through inhibition of STAT3 activity. *PloS one* 8**,** e61411.

Langmead, B., and Salzberg, S.L. (2012). Fast gapped-read alignment with Bowtie 2. *Nat Methods* 9**,** 357-359.

Li, H., Handsaker, B., Wysoker, A., Fennell, T., Ruan, J., Homer, N., Marth, G., Abecasis, G., Durbin, R., and Genome Project Data Processing, S. (2009). The Sequence Alignment/Map format and SAMtools. *Bioinformatics* 25**,** 2078-2079.

Liao, X., Sharma, N., Kapadia, F., Zhou, G., Lu, Y., Hong, H., Paruchuri, K., Mahabeleshwar, G.H., Dalmas, E., Venteclef, N., Flask, C.A., Kim, J., Doreian, B.W., Lu, K.Q., Kaestner, K.H., Hamik, A., Clement, K., and Jain, M.K. (2011). Kruppel-like factor 4 regulates macrophage polarization. *The Journal of clinical investigation* 121**,** 2736-2749.

Martin, M. (2011). Cutadapt removes adapter sequences from high-throughput sequencing reads. *EMBnet.journal* 17**,** 10-12.

Min, B.K., Suk, K., and Lee, W.H. (2013). Stimulation of CD107 affects LPS-induced cytokine secretion and cellular adhesion through the ERK signaling pathway in the human macrophage-like cell line, THP-1. *Cellular immunology* 281**,** 122-128.

Ni Gabhann, J., Hams, E., Smith, S., Wynne, C., Byrne, J.C., Brennan, K., Spence, S., Kissenpfennig, A., Johnston, J.A., Fallon, P.G., and Jefferies, C.A. (2014). Btk regulates macrophage polarization in response to lipopolysaccharide. *PloS one* 9**,** e85834.

Oh, D.Y., Morinaga, H., Talukdar, S., Bae, E.J., and Olefsky, J.M. (2012). Increased macrophage migration into adipose tissue in obese mice. *Diabetes* 61**,** 346-354.

Robinson, M.D., Mccarthy, D.J., and Smyth, G.K. (2010). edgeR: a Bioconductor package for differential expression analysis of digital gene expression data. *Bioinformatics* 26**,** 139-140.

Robinson, M.D., and Oshlack, A. (2010). A scaling normalization method for differential expression analysis of RNA-seq data. *Genome Biol* 11**,** R25.

Trapnell, C., Pachter, L., and Salzberg, S.L. (2009). TopHat: discovering splice junctions with RNA-Seq. *Bioinformatics* 25**,** 1105-1111.

Trapnell, C., Williams, B.A., Pertea, G., Mortazavi, A., Kwan, G., Van Baren, M.J., Salzberg, S.L., Wold, B.J., and Pachter, L. (2010). Transcript assembly and quantification by RNA-Seq reveals unannotated transcripts and isoform switching during cell differentiation. *Nat Biotechnol* 28**,** 511-515.

Weischenfeldt, J., and Porse, B. (2008). Bone Marrow-Derived Macrophages (BMM): Isolation and Applications. *CSH protocols* 2008**,** pdb prot5080.

Yates, A., Akanni, W., Amode, M.R., Barrell, D., Billis, K., Carvalho-Silva, D., Cummins, C., Clapham, P., Fitzgerald, S., Gil, L., Giron, C.G., Gordon, L., Hourlier, T., Hunt, S.E., Janacek, S.H., Johnson, N., Juettemann, T., Keenan, S., Lavidas, I., Martin, F.J., Maurel, T., Mclaren, W., Murphy, D.N., Nag, R., Nuhn, M., Parker, A., Patricio, M., Pignatelli, M., Rahtz, M., Riat, H.S., Sheppard, D., Taylor, K., Thormann, A., Vullo, A., Wilder, S.P., Zadissa, A., Birney, E., Harrow, J., Muffato, M., Perry, E., Ruffier, M., Spudich, G., Trevanion, S.J., Cunningham, F., Aken, B.L., Zerbino, D.R., and Flicek, P. (2016). Ensembl 2016. *Nucleic Acids Res* 44**,** D710-716.
